# Molecular Mapping and QTL for Expression Profiles of Flavonoid Genes in *Brassica napus*

**DOI:** 10.3389/fpls.2016.01691

**Published:** 2016-11-09

**Authors:** Cunmin Qu, Huiyan Zhao, Fuyou Fu, Kai Zhang, Jianglian Yuan, Liezhao Liu, Rui Wang, Xinfu Xu, Kun Lu, Jia-Na Li

**Affiliations:** ^1^Chongqing Engineering Research Center for Rapeseed, College of Agronomy and Biotechnology, Southwest UniversityChongqing, China; ^2^Engineering Research Center of South Upland Agriculture of Ministry of Education, Southwest UniversityChongqing, China; ^3^Department of Botany and Plant Pathology, Purdue UniversityWest Lafayette, IN, USA

**Keywords:** *Brassica napus*, expression profiles, flavonoid genes, genetic map, QTLs

## Abstract

Flavonoids are secondary metabolites that are extensively distributed in the plant kingdom and contribute to seed coat color formation in rapeseed. To decipher the genetic networks underlying flavonoid biosynthesis in rapeseed, we constructed a high-density genetic linkage map with 1089 polymorphic loci (including 464 SSR loci, 97 RAPD loci, 451 SRAP loci, and 75 IBP loci) using recombinant inbred lines (RILs). The map consists of 19 linkage groups and covers 2775 cM of the *B. napus* genome with an average distance of 2.54 cM between adjacent markers. We then performed expression quantitative trait locus (eQTL) analysis to detect transcript-level variation of 18 flavonoid biosynthesis pathway genes in the seeds of the 94 RILs. In total, 72 eQTLs were detected and found to be distributed among 15 different linkage groups that account for 4.11% to 52.70% of the phenotypic variance atrributed to each eQTL. Using a genetical genomics approach, four eQTL hotspots together harboring 28 eQTLs associated with 18 genes were found on chromosomes A03, A09, and C08 and had high levels of synteny with genome sequences of *A. thaliana* and Brassica species. Associated with the *trans*-eQTL hotspots on chromosomes A03, A09, and C08 were 5, 17, and 1 genes encoding transcription factors, suggesting that these genes have essential roles in the flavonoid biosynthesis pathway. Importantly, *bZIP25*, which is expressed specifically in seeds, *MYC1*, which controls flavonoid biosynthesis, and the R2R3-type gene *MYB51*, which is involved in the synthesis of secondary metabolites, were associated with the eQTL hotspots, and these genes might thus be involved in different flavonoid biosynthesis pathways in rapeseed. Hence, further studies of the functions of these genes will provide insight into the regulatory mechanism underlying flavonoid biosynthesis, and lay the foundation for elaborating the molecular mechanism of seed coat color formation in *B. napus*.

## Introduction

*Brassica napus* L. (2*n* = 38, AACC) is an economically important oilseed crop that is widely cultivated as a source of vegetable oil, biodiesel, and protein-rich meal for animal feed (Kimber and Mcgregor, 1995). Yellow *B. napus* seeds are the most desirable, as they have thinner seed coats and higher seed oil and protein contents than do the dark-seeded varieties with a similar genetic background (Olsson, 1960; Tang et al., 1997; Meng et al., 1998). Several studies have shown that seed coat color is determined by the content of the phenolic compounds cyanidin and procyanidin in *B. napus* (Marles and Gruber, 2004; Lepiniec et al., 2006; Qu et al., 2013). These pigments are mainly composed of polymers of proanthocyanidin (PA), which is synthesized via the flavonoid-anthocyanin-proanthocyanidin pathway (simplified as flavonoid pathway here), a core branch of the phenylpropanoid pathway (Bharti and Khurana, 2003; Gachon et al., 2005). In *A. thaliana*, most of the structural and regulatory loci of the core flavonoid biosynthesis pathway have been cloned and functionally characterized, and over 22 Arabidopsis mutants (*tt1*–*tt19, ttg1, ttg2*, and *aha10*) with altered patterns of seed coat color have been identified. Loss-of-function mutations [*tt* (transparent testa) or *tt-like*] in any one of these single-copy loci change the seed coat color from dark brown to yellow (Wan et al., 2002; Winkel-Shirley, 2002; Baudry et al., 2004; Lepiniec et al., 2006). In addition, members of the MYB and R/B-like basic helix-loop-helix (bHLH) families were demonstrated to be involved in the flavonoid biosynthesis pathway; for example, a transcriptional activation MYB-bHLH-WD40 complex (MBW) consisting of R2R3 MYB, bHLH, and WD40 proteins was found to be directly involved in the regulation of anthocyanin biosynthetic genes and the bHLH proteins were found to play essential roles in the synergistic regulation of flavonid accumulation (Baudry et al., 2006; Dubos et al., 2008; Kitamura et al., 2010; Stracke et al., 2010). Furthermore, *TT2* (R2R3-MYB), *TT8* (bHLH), and *TTG1* (WDR) affect the production of PA, which is a substrate of the flavonoid pathway (Baudry et al., 2004, 2006; Lepiniec et al., 2006), and *AtMYB4*, bHLH*IN1*, and *AtICX1* regulate various flavonoid biosynthesis pathways (Burr et al., 1996; Jin et al., 2000; Wade et al., 2003). Moreover, some homologs of genes involved in flavonoid biosynthesis have been cloned and characterized in *B*. *napus* (Wei et al., 2007; Xu et al., 2007; Ni et al., 2008; Akhov et al., 2009; Auger et al., 2009; Chai et al., 2009; Lu et al., 2009; Chen et al., 2013). These results provide a foundation for further studies of the molecular and regulatory mechanisms underlying seed coat color formation in *B. napus*. Based on linkage mapping with DH, RIL, and F2 *B. napus* populations, a major QTL was identified on Chr. A09 that accounted for 40–60% of the phenotypic variance of seed coat color (Somers et al., 2001; Liu et al., 2005; Badani et al., 2006; Fu et al., 2007; Xiao et al., 2007; Rahman et al., 2010; Zhang et al., 2011). Candidate genes involved in seed coat color determination, such as *TT10* and *AHA10*, have still not successfully been used in rapeseed breeding programs aimed at producing seeds with a particular coat color (Fu et al., 2007; Stein et al., 2013; Zhang et al., 2013). Efforts to breed yellow-seeded *B. napus* have been largely unsuccessful, since seed coat color is a typical quantitative trait under polygenic control (Rahman, 2001; Liu et al., 2005; Badani et al., 2006) that is influenced by factors such as maternal effects and the environment (Deynze et al., 1993). Hence, the molecular mechanism underlying yellow seed coat formation in *Brassica* is poorly understood.

Previous research suggested that one to four genes determine seed coat color in *B. napus* (Somers et al., 2001; Xiao et al., 2007; Zhang et al., 2011). Further, traditional studies for mapping quantitative trait loci (QTLs) had focused on identifying the major QTLs associated with seed coat color in different populations (Liu et al., 2005, 2006; Badani et al., 2006; Fu et al., 2007; Xiao et al., 2007; Yan et al., 2009; Zhang et al., 2011). However, these genes remain to be cloned and functionally characterized. Recently, the genome of the allopolyploid *B. napus* was released, and a total of 1097 and 1132 genes were annotated on the An and Cn subgenomes, respectively (Chalhoub et al., 2014). Moreover, genome-wide gene expression profiling has been extensively used to generate biological hypotheses based on differential expression. mRNAs that are differentially expressed among individuals can be considered as quantitative traits and their variation can be used to map expression quantitative trait loci (eQTLs) (Jansen and Nap, 2001). Based on the location of the eQTL relative to the location of the affected gene(s), each locus can be classified as *cis* acting (i.e., eQTL located near the affected gene) or *trans* acting (i.e., eQTL does not coincide with the affected gene) (Deutsch et al., 2005; Doss et al., 2005; Hubner et al., 2006). Therefore, this approach not only detects the expression of a specific gene and the genotype at that gene's locus, but it also reveals clustered *trans*-eQTLs that are simultaneously regulated by a large fraction of the transcriptome (Brem et al., 2002; Schadt et al., 2003; Morley et al., 2004). This approach has been successfully used in crop plants to detect transcript-level variation and downstream phenotypic trait variation (Jordan et al., 2007; Shi et al., 2007; West et al., 2007; Potokina et al., 2008; Xiao et al., 2013, 2014; Del Carpio et al., 2014; Basnet et al., 2015, 2016). Although eQTLs have successfully been cloned in plants (Werner et al., 2005; Zhang et al., 2006), global eQTL analysis in a large mapping population of plants has not hitherto been performed.

Here, we greatly increased the marker density of a RIL genetic map in rapeseed, from 420 loci with a total length of 1744 cM (Fu et al., 2007) to 1089 loci with a total length of 2775 cM. To decipher the upstream regulatory network underlying flavonoid biosynthesis, we used a sample of 94 recombinant inbred lines (RILs) from a population derived from a cross between the female parent GH06 and the male parent ZY821. The transcript levels of 18 flavonoid biosynthesis pathway genes were evaluated using RNA extracted from seeds of the RIL population at 30 days after flowering (DAF). Regarding the expressed transcript level of each gene in the RILs as a quantitative trait, we then performed eQTL analysis to detect eQTLs. Using this method, we were able to construct the regulatory pathway that contributes to the complex trait of seed coat color. We thus demonstrate that eQTL mapping can be successfully applied to *B. napus*.

## Materials and methods

### Plant materials and total RNA extraction

The recombinant inbred line (RIL) population was derived from a cross between the male parent Zhongyou 821 and the female parent GH06 followed by 10 successive generations of selfing by single seed propagation. Parental lines and RILs were sown in field trials at the plant breeding station at the Chongqing Rapeseed Technology Research Center (CRTRC) in 2012, as previously described (Fu et al., 2007). The seeds of 94 F_2:10_ RILs were harvested at 30 days after flowering (DAF) and used for total RNA isolation. Total RNA was extracted using the Plant RNA Mini Kit (Watson Biotechnologies, Inc., China). To remove contaminating genomic DNA, the total RNA was treated with RNase-free DNase I (TaKaRa, China). The quality and concentration of total RNA samples were assessed by agarose gel electrophoresis and spectrophotometry.

### SSR marker assays

A total of 1850 SSR markers were developed to increase the density of the genetic map, including 1014 new developmental SSR markers (Supplementary Table S6), according to the *B. rapa* and *B. oleracea* genome (prefixed by “SWUA” and “SWUC,” respectively), 259 published SSR markers (Landry et al., 1991; Ferreira et al., 1994; Foisset et al., 1995; Uzunova et al., 1995; Lombard and Delourme, 2001; Xu et al., 2001; Zhao and Meng, 2003; Liu et al., 2005; Piquemal et al., 2005; Qiu et al., 2006; Fu et al., 2007; Radoev et al., 2008; Cheng et al., 2009; Kim et al., 2009), 447 SSR markers, and 130 intron-based polymorphism (IBP) markers provided by Dr. Beom-Seok Park and Dr. Soo-Jin Kwon of the National Academy of Agricultural Science (South Korea) (prefixed by “KC-,” “KR-,” “KA-,” “KS-,” “H-,” “B-,” and “S-”) and by Dr. Jingling Meng (Huazhong Agricultural University). Genomic DNA was extracted from the young leaves of five pooled plants per genotype using a standard CTAB extraction protocol.

PCR reactions were performed in 96-well plates in a volume of 10 μL. The composition of the mixture was as follows: 20 ng/μl of DNA template, 0.5 pmol of each primer, 0.2 mM dNTP mix, 1 mM MgCl_2_, 10 × PCR reaction buffer (with 15 mM MgCl_2_, *TransGen* Biotech), and 0.5 units of Taq DNA polymerase (*TransGen* Biotech). PCR was carried out in PTC-100 and PTC-200 thermocyclers with the following program (slightly modified from that of Piquemal et al., 2005): 94°C for 4 min; 35 cycles consisting of denaturation at 94°C for 45 s, annealing at 55°C for 45 s, and elongation at 72°C for 1 min; then a final elongation at 72°C 10 min. All PCR products were detected using non-denaturing polyacrylamide gel electrophoresis (10% polyacrylamide) on a DYCZ-30 electrophoresis gel with silver staining (Zhang et al., 2002).

### Mapping and alignments

All markers were tested for Mendelian segregation ratios using the Chi-square (χ^2^) test for goodness of fit with the expected 1:1 (*a* ≥ 0.05) ratio of individual markers in a RIL population. JoinMap 4.0 was used to build a high-density genetic linkage map with a minimum logarithm of odds score of 3.0. Genetic distances were calculated according to the Kosambi formula (Kosambi, 1944). To reconcile the linkage maps with Brassica and *A. thaliana* chromosomes, the genetic map was aligned with their pseudo-chromosomes using the base-sequences of each primer (Supplementary Table S3). Intron-based polymorphism (IBP) markers were developed directly from scaffold sequences, and the SSRs were considered anchored if the sequence of both primers matched the genome sequences (85% overlap and 98% identity). Similarly, the unigene sequences containing SSRs were aligned with *A. thaliana* genomic sequences using BLASTN. Sequences were regarded as homologs of loci in the *A. thaliana* genome if they had an *e*-value threshold of ≤ 1e−10. Regions that had conserved collinearity with *A. thaliana* were regarded as homologous syntenic regions.

### Quantitative real-time polymerase chain reaction analysis

One microgram of each RNA sample was used to make first-strand cDNA in a 20 μl reaction with Oligo dT-Adaptor Primer using the RNA PCR Kit (AMV) Ver. 3.0 (TaKaRa, China). Primers for amplifying partial sequences of genes involved in the flavonoid biosynthesis pathway were designed from conserved nucleotide regions identified by multiple alignments of sequences from *A. thaliana* (http://www.arabidopsis.org/) and *B. napus* (Chalhoub et al., 2014; http://www.genoscope.cns.fr/brassicanapus/). Primers of genes for real-time PCR are listed in Supplementary Table S1. Real-time PCR was conducted using SYBR® Premix Ex Taq™ II (Perfect Real Time) (TaKaRa, China) in a PCR mixture consisting of 10 μl SYBR® Premix Ex *Taq*™ II, 1 to 5 μl of template cDNA, 0.8 μM of each PCR primer, and ddH_2_O to a final volume of 20 μl. Cycling conditions were 95°C for 2 min, followed by 40 cycles at 95°C for 10 s and 60°C for 20 s, and a dissociation curve consisting of a 10-s incubation at 95°C, 5-s incubation at 65°C, and a ramp up to 95°C, and amplifications were run on the Bio-Rad CFX96 Real Time System (USA). Melting curves were used to validate product specificity. The relative expression of the target genes was analyzed with the 2^−ΔΔCt^ method (Supplementary Table S7) using *BnACTIN7* (EV116054) and *BnUBC21* (EV086936) as the internal controls (Wu et al., 2010). All samples were amplified in triplicate and used for the total RNA preparation. All qRT-PCR assays were repeated three times, and the mean value was used for further analysis. The Pearson correlation coefficient (*r*) and probability value (*p*) were used to display correlations and the significance of differences in expression between any two genes using SPSS 13.0. A probability value of *p* < 0.05 was considered to indicate statistical significance.

### Expression profiles of QTLs for genes associated with the flavonoid biosynthesis pathway

The eQTLs for each gene were estimated by the composite interval method (CIM) with WinQTL Cartographer 2.5 software (Lander and Botstein, 1989; Wang et al., 2006). CIM was used to scan the genetic map and estimate the likelihood of a QTL and its corresponding effect at every 1 cM. A LOD (Log likelihood) of ≥2.5 indicated that the highest LOD score position in the interval was a QTL for a trait. The relative contribution of a genetic component was calculated as the proportion of the additive effect and phenotypic variance explained by that component. The linkage group order and QTLs in the map were processed using Mapchart 2.1 (Voorrips, 2002). QTL nomenclature, following a previously described system (Mccouch et al., 1997), started with “*q*” and was followed by an abbreviation of the trait name, the name of the linkage group, and the number of eQTLs in the linkage group that affect the trait. For instance, “*qBAN-4-1*” denotes the first eQTL associated with *BAN* expression and is detected and located on the fourth linkage group.

### Analysis of sequences flanking *trans*-eQTLs

To determine the location of flavonoid biosynthesis pathway genes on *B. napus* chromosomes and to establish the type of eQTL, the cDNA sequences of orthologous genes in Arabidopsis and sequences of eQTL markers were used as query for a BLASTN search against the *B. napus* “Darmor-Bzh” reference genome (Cheng et al., 2014). The 200-kb sequences flanking each marker in *B. napus* were extracted from the reference genome. Genes in these flanking sequences were identified and annotated. *cis*-eQTLs coincide with the location of the underlying gene, whereas *trans*-eQTLs do not, implying that the observed eQTL represents the position of a locus that controls the expression variation of the target gene.

## Results

### Analysis of expression levels of 18 genes involved in flavonoid biosynthesis

**Figure 1:**
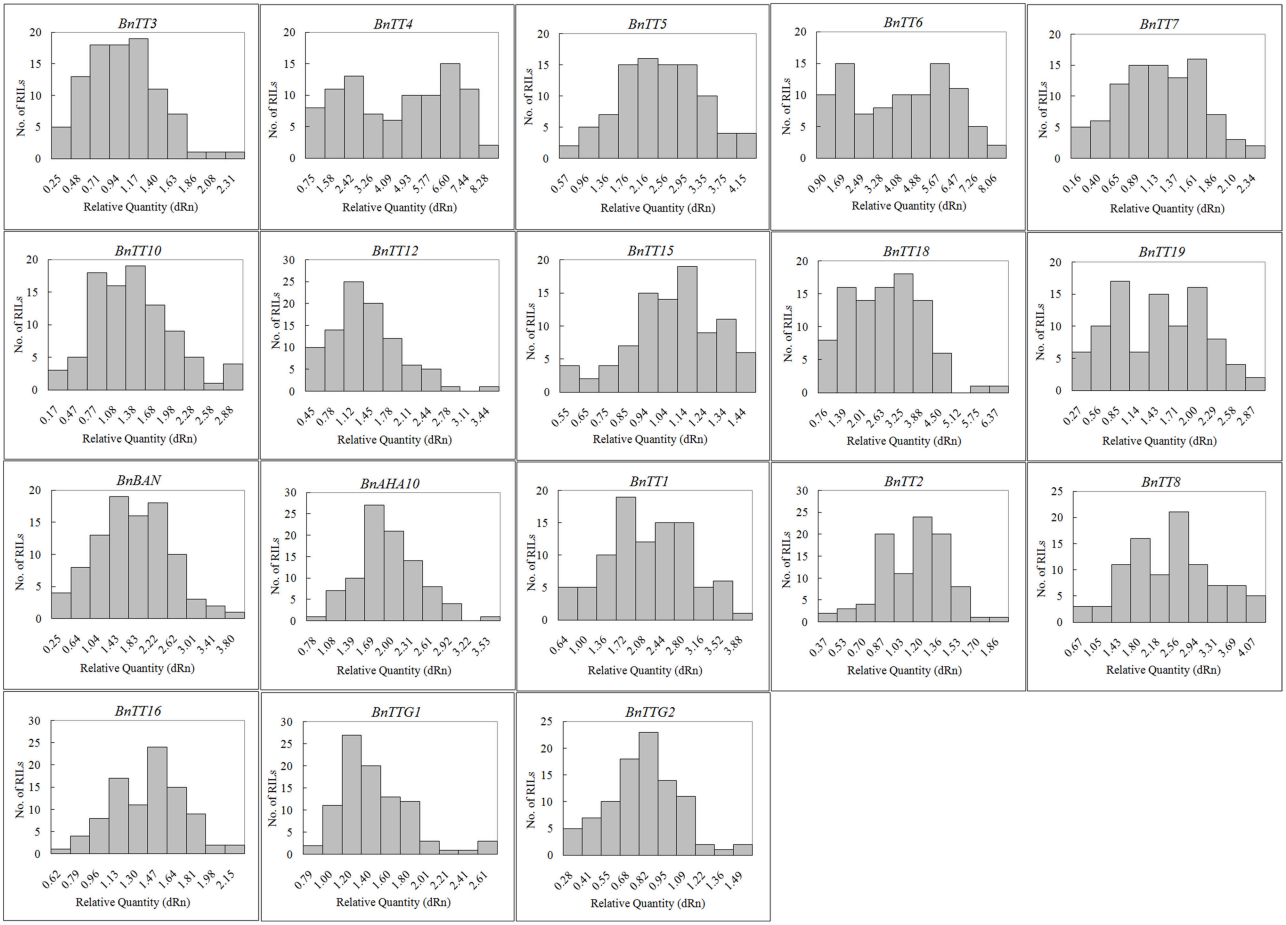
**The frequency distribution of relative expression levels of flavonoid biosynthesis genes in ***B. napus*****. Abscissa: Relative expression level of each gene, Ordinate: The number of lines.

We assayed the expression levels of 18 flavonoid biosynthesis genes (Supplementary Figure S3), including 12 structural genes (i.e., *BnTT3, BnTT4, BnTT5, BnTT6, BnTT7, BnTT10, BnTT12, BnTT15, BnTT18, BnTT19, BnAHA10*, and *BnBAN*) and six regulatory genes (*BnTT1, BnTT2, BnTT8, BnTT16, BnTTG1*, and *BnTTG2*) (Qu et al., 2013) in *B. napus* RILs derived from a cross between the male parent Zhongyou 821 and female parent GH06 by qRT-PCR, and normalized the gene expression levels according to the expression values of the male parent ZY821. We observed significant differences in the expression levels of these 18 genes between the parental lines and RILs (*p* < 0.01 or *p* < 0.05, Supplementary Table S2). Both skewness and kurtosis in absolute values implied that the expression levels of these genes had a normal distribution in the RILs, and that the expression levels were distributed continuously, as expected for a quantitative trait (Figure 1). In addition, the expression levels of all pairwise combinations of these 18 genes were subjected to correlation analysis, and significant positive and negative correlations were detected between the expression levels of gene pairs (Table 1), in accordance with their common function in the flavonoid biosynthesis pathway. For example, *BnTT4* and *BnTT5* catalyze the production of the precursor of all flavonoids and *BnTT6* and *BnTT3* convert naringenin into leucocyanidin and leucopelargonidin, respectively (Pelletier and Shirley, 1996; Burbulis and Winkel-Shirley, 1999; Abrahams et al., 2003; Kasai et al., 2007). Therefore, significant positive correlations were found among these genes (Table 1), but they exhibited a significant negative correlation with *BnTT7* (Table 1), which encodes an enzyme that converts dihydrokaempferol into dihydroquercetin in the flavonoid biosynthesis pathway (Schoenbohm et al., 2000), suggesting that there is competition for catalyzing the same precursors of the flavonoid biosynthesis pathway. Furthermore, the expression of these genes was significantly positively correlated with that of structural (*BnTT12, BnTT18*, and *BnAHA10*) and regulatory (*BnTT1, BnTT8*, and *BnTTG1*) genes associated with flavonoid biosynthesis (Table 1), indicating that these genes are determined by a common upstream gene or activated by the same biosynthetic precursors of flavonoid in the biosynthesis pathway.

**Table 1:** **Correlation coefficient among relative expression levels of all flavonoid genes in the RIL populations**.

**Name**	***BnTT3***	***BnTT4***	***BnTT5***	***BnTT6***	***BnTT7***	***BnTT10***	***BnTT12***	***BnTT15***	***BnTT18***	***BnTT19***	***BnBAN***	***BnAHA10***	***BnTT1***	***BnTT2***	***BnTT8***	***BnTT16***	***BnTTG1***
*BnTT4*	0.819^**^																
*BnTT5*	0.743^**^	0.707^**^															
*BnTT6*	0.856^**^	0.910^**^	0.755^**^														
*BnTT7*	−0.358^**^	−0.289^**^	−0.515^**^	−0.296^**^													
*BnTT10*	−0.076	−0.080	−0.217^*^	−0.052	0.283^**^												
*BnTT12*	0.829^**^	0.844^**^	0.732^**^	0.890^**^	−0.357^**^	−0.095											
*BnTT15*	0.143	0.062	0.192	0.062	0.006	0.066	0.101										
*BnTT18*	0.782^**^	0.849^**^	0.642^**^	0.906^**^	0.237^*^	0.054	0.832^**^	0.004									
*BnTT19*	−0.634^**^	−0.710^**^	−0.539^**^	−0.750^**^	−0.086	0.042	−0.635^**^	0.027	−0.760^**^								
*BnBAN*	−0.686^**^	−0.744^**^	−0.594^**^	−0.750^**^	0.139	0.004	−0.708^**^	−0.119	−0.762^**^	0.773^**^							
*BnAHA10*	0.583^**^	0.632^**^	0.585^**^	0.660^**^	−0.288^**^	−0.138	0.683^**^	0.287^**^	0.639^**^	−0.406^**^	−0.450^**^						
*BnTT1*	0.791^**^	0.850^**^	0.679^**^	0.872^**^	−0.273^**^	−0.064	0.874^**^	0.079	0.855^**^	−0.665^**^	−0.723^**^	0.643^**^					
*BnTT2*	0.168	0.170	0.196	0.125	−0.080	−0.094	0.311^**^	0.258^*^	0.101	−0.040	−0.115	0.339^**^	0.242^*^				
*BnTT8*	0.721^**^	0.687^**^	0.686^**^	0.704^**^	−0.354^**^	−0.075	0.718^**^	0.234^*^	0.767^**^	−0.516^**^	−0.622^**^	0.606^**^	0.748^**^	0.23^*^			
*BnTT16*	−0.144	−0.023	0.124	−0.085	−0.124	−0.385^**^	−0.017	0.462^**^	−0.063	0.154	0.093	0.271^**^	−0.028	0.225^*^	0.053		
*BnTTG1*	0.290^**^	0.320^**^	0.248^*^	0.324^**^	−0.050	0.075	0.309^**^	0.259^*^	0.337^**^	−0.168	−0.158	0.513^**^	0.321^**^	0.213^*^	0.335^**^	0.153	
*BnTTG2*	0.175	0.062	0.109	0.043	−0.005	0.162	0.060	0.381	0.032	0.098	−0.031	0.259^*^	0.036	0.144	0.264^*^	0.101	0.237

*, ** *Correlation is significant based on Student's t-test: P < 0.05 and P < 0.01, respectively*.

### Linkage map construction and alignments

**Figure 2:**
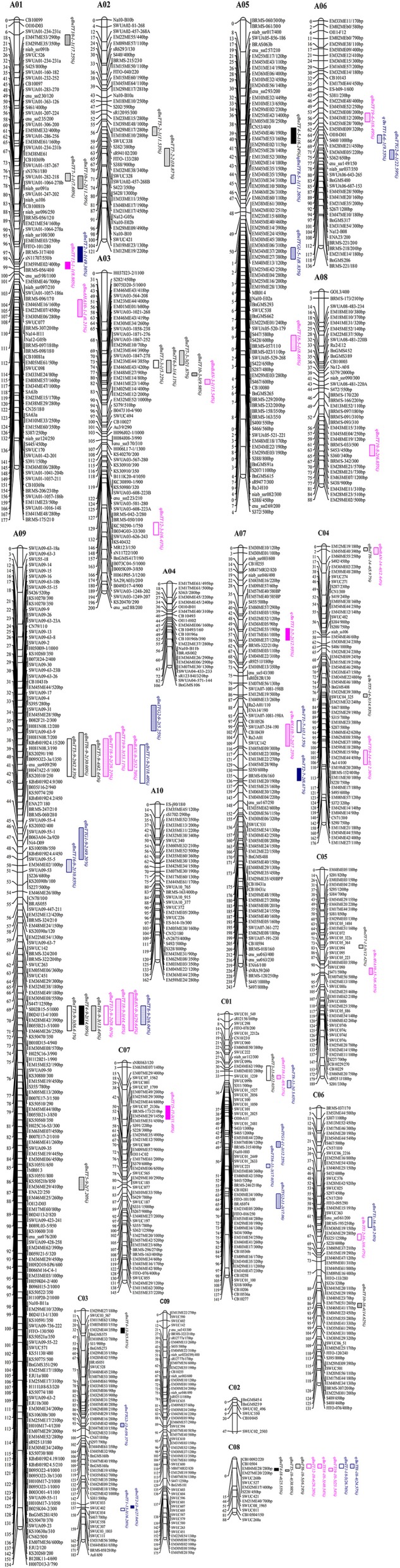
**Linkage map of ***B. napus*** and eQTL detection for flavonoid biosynthesis genes in ***B. napus*****. The QTLs and markers were drawn using MapChart Version 2.0 software (Voorrips, 2002). The distances (in centiMorgan, cM) to the left of each linkage group were calculated using the Kosambi function.

A total of 1087 molecular markers, including 464 SSRs, 97 RAPDs, 451 SRAPs, and 75 IBP, were mapped on 19 linkage groups, covering 2, 775 cM of the *B. napus* genome, according to the Kosambi function previously published (Fu et al., 2007) (Figure 2). The average distance between two adjacent markers was 2.55 cM. The number of markers per linkage group varied from 6 to 184, and the length of each linkage group varied from 47.22 to 243.46 cM, with an average genetic distance of 0.83 cM on chromosome A09 and 7.87 cM on chromosome C02 (Table 2, Figure 2). Nineteen linkage groups were assigned to the public linkage maps based on anchored SSR markers. The results showed that the order of markers was relatively consistent with those in published maps (Piquemal et al., 2005; Radoev et al., 2008; Cheng et al., 2009; Kim et al., 2009; Xu et al., 2010). The number of anchored markers per chromosome ranged from 0 (C06) to 84 (A09), with an average of 12.47 for the 237 public markers evaluated, and from 2 (A04, A06) to 21 (A02), with an average of 10.32 for the 196 specific markers newly developed from the *B. rapa* and *B. oleracea* genomes. However, 13 interval gaps in which adjacent markers were separated by >15 cM were distributed on chromosomes A02, A03, A04, A06, A10, C01, C02, C04, C05, and C08, respectively (Table 2, Figure 2). These results show that the 19 linkage groups included in our linkage map have strong homology within particular linkage groups, and could be universally used in *B. napus* research.

**Table 2:** **Distribution of molecular markers on different linkage groups**.

**Linkage group**	**No. of loci**	**No of intervals^a^**	**No. of gaps^b^**	**Average interval (cM)**	**Length (cM)**	**No. of anchored markers**	**No. of specific primers from *B. rapa* and *B. oleracea***
A01	87	49	0	2.03	176.56	8	6
A02	41	30	1	2.95	120.91	20	21
A03	62	44	1	3.23	200.32	30	14
A04	23	13	2	4.63	106.49	9	2
A05	84	27	0	1.36	114.45	16	4
A06	44	37	1	3.09	136.13	13	2
A07	82	65	0	2.97	243.47	15	6
A08	38	30	0	2.31	87.71	12	3
A09	184	47	0	0.83	152.74	84	47
A10	35	29	1	4.63	161.88	3	6
C01	61	32	1	2.31	140.68	9	18
C02	6	3	1	7.87	47.22	3	3
C03	53	38	0	3.45	182.65	5	9
C04	60	39	1	2.94	176.18	1	4
C05	45	33	1	4.19	188.61	1	10
C06	61	42	0	2.15	131.32	0	6
C07	49	35	0	2.97	145.43	2	11
C08	14	3	3	6.29	88.02	2	6
C09	58	40	0	3.01	174.51	4	18
Total	1087	636	13	2.55	2775	237	196

a distance between adjacent markers > 1 cM;

b *distance between adjacent markers > 15 cM*.

We identified 531 pairs of sequence-informative markers and mapped these markers to 19 linkage groups (Figure 2). Of these, 370 were anchored to the A and C sub-genomes of *B. rapa* and *B. oleracea*, which have high levels of nucleotide sequence similarity (*E*-value ≤ 1e-10), and 21 were mapped to two or three loci (Supplementary Table S3) that had high levels of sequence similarity with sequences in *B. rapa* (Supplementary Figure S1) and *A. thaliana* (Supplementary Figure S2). However, the relative position of some markers was inconsistent between the linkage map of *B. napus* and the physical map of *B. rapa* (Supplementary Figure S1), possibly due to genomic rearrangement events such as inversions and intra-chromosomal translocations and discrepancies related to different population sizes being used for mapping in the two species (Jiang et al., 2011). These results can be used to identify candidate genes involved in the flavonoid biosynthesis pathway based on the *B. napus* “Darmor-Bzh” reference genome (Chalhoub et al., 2014; http://www.genoscope.cns.fr/brassicanapus/) and The Arabidopsis Information Resource (TAIR, http://www.arabidopsis.org/index.jsp).

### eQTL analysis of 18 genes involved in flavonoid biosynthesis

**Figure 3:**
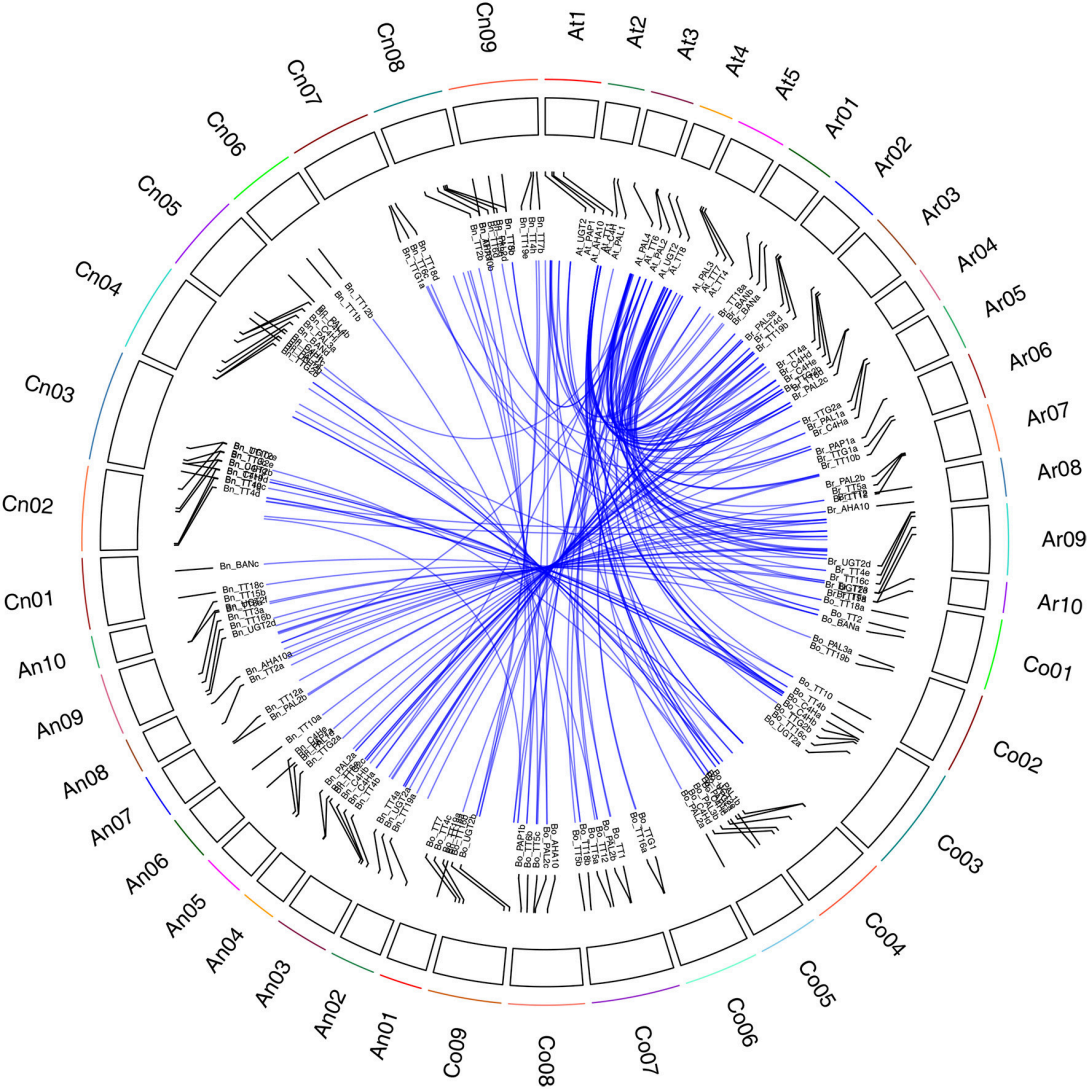
**Syntenic relationship of flavonoid biosynthesis genes between ***A. thaliana*** and ***Brassica*** genomes**. Black frame with different colors represents chromosomes of four species. Ar01 ~ Ar10 represent pseudo-chromosomes of the *B. rapa* genome, Co01 ~ Co09 represent pseudo-chromosomes of the *B. oleracea* genome, An01 ~ An10 and Cn01 ~ Cn09 represent pseudo-chromosomes of the *B. napus* genome, and At1 ~ At5 represent chromosomes of the *A. thaliana* genome. Blue lines represent the relationship between orthologous gene pairs from different species.

In an analysis of orthologous regions of eQTLs, we identified 243 copies of 18 genes involved in flavonoid biosynthesis from *A. thaliana* (37), *B. rapa* (55), *B. oleracea* (52), and *B. napus* (99) (Supplementary Table S4; Figure 3) (Krzywinski et al., 2009), respectively. Seventy-two eQTLs for 18 flavonoid biosynthesis pathway genes were detected and found to be distributed among 15 different linkage groups, with 3 to 5 eQTLs per gene. Each eQTL could explain 4.11–52.70% of the phenotypic variance (Table 3, Figure 2). The results are consistent with sequences present as a single copy in the *A. thaliana* genome being present as 2–8 copies in *B. napus* (Cavell et al., 1998). Moreover, four eQTL hotspots were identified on chromosomes A03, A09, and C08, including 28 eQTLs for 12 genes. According to the value of additive effects, the positive alleles of 23 eQTLs for seven genes were derived from the male parent ZY821, whereas the remaining five eQTLs (i.e., *qBnTT5-18-4, qBnTT7-3-3, qBnTT7-9-4, qBnTT18-18-5*, and *qBnTT19-18-5)* were derived from the female parent GH06 (Table 3). Furthermore, two eQTL hotspots were located up- and down-stream of the major QTL region (32–36 cM of chromosome A09) for seed coat color, between regions 18–22 cM and 72–76 cM of chromosome A09, respectively. In addition, 22 major eQTLs explaining over 20% of the total phenotypic variation were found to be located on chromosomes A01, A03, A06, A09, C03, and C08 (Figure 2). Their positive alleles were derived from both of the parents.

**Table 3:** **eQTLs for flavonoid biosynthetic pathway genes detected from the ***B. napus*** RIL population**.

**QTL name**	**Chr**.	**Marker-Interval^a^**	**Position**	**LOD**	**Add.^b^**	***R^2^*^c^**
*qBnTT3-3-1*	A03	EM01ME01/b80bp–EM46ME43/419bp	58.15	2.59	−0.46	5.22
*qBnTT3-9-2*	A09	SWUA09-55-15–SWUA09-2	21.54	13.69	−1.21	42.83
*qBnTT3-9-3*	A09	KS50470(R09)/350–KS30880(A09)/300	75.24	9.42	−1.08	36.61
*qBnTT3-11-4*	C01	SWUC099a(C01)–SWUC01_1527	33.29	3.18	0.48	12.03
*qBnTT4-5-1*	A05	EM36ME06/400bp–cnu_ssr293/200	28.97	3.55	0.38	6.24
*qBnTT4-9-2*	A09	B010D15-4(A09)/940–SWUA09-50	74.04	3.44	−0.88	14.09
*qBnTT4-13-3*	C03	SWUC03_567–EM11ME62/130bp	17.87	2.82	−0.38	5.81
*qBnTT4-18-4*	C08	SWUC421(C03/C08)–EM21ME40/700bp	18.37	4.61	−0.91	25.55
*qBnTT5-2-1*	A02	SWUC338(C04/C09)–FITO-133/280	63.34	4.55	−0.30	11.53
*qBnTT5-9-2*	A09	KS10591(R09)350–KS50521a(R09)/350	99.69	2.88	−0.23	7.20
*qBnTT5-14-3*	C04	EM60ME42/620bp–EM42ME37/100bp	95.85	4.30	−0.33	14.93
*qBnTT5-18-4*	C08	SWUC421(C03/C08)–EM21ME40/700bp	18.37	7.10	0.54	38.98
*qBnTT6-3-1*	A03	EM01ME01/b80bp–EM46ME43/419bp	58.15	2.56	−0.54	4.11
*qBnTT6-9-2*	A09	SWUA09-55-15–SWUA09-2	21.54	13.64	−1.53	30.00
*qBnTT6-9-3*	A09	B010D15-4(A09)/940–KS30880(A09)/300	73.94	6.24	−0.65	13.64
*qBnTT6-14-4*	C04	EM12ME19/180bp–EM45ME40/390bp	1.01	2.59	−0.29	5.97
*qBnTT6-18-5*	C08	SWUC527(C08)–SWUC421(C03/C08)	10.01	4.30	−0.57	16.28
*qBnTT7-1-1*	A01	EM58ME32/400bp–EM38ME61/160bp	62.09	2.91	−0.30	7.60
*qBnTT7-2-2*	A02	SWUC328(C03/C09)–EM48ME17/190bp	70.99	6.74	−0.62	18.87
*qBnTT7-3-3*	A03	SWUA03-564-208–SWUA03-1021-268	50.96	8.04	0.65	21.87
*qBnTT7-9-4*	A09	SWUA09-55-15–SWUA09-2	21.54	14.30	1.43	42.64
*qBnTT7-15-5*	C05	SWUC072(C05) –SWUC05_364	69.03	4.97	0.50	12.42
*qBnTT10-1-1*	A01	SWUA01-234-231c–EM47ME53/290bp	8.30	2.75	−0.55	17.25
*qBnTT10-1-2*	A01	SWUA01-286-256–EM38ME61/400bp	64.68	4.01	−0.35	11.59
*qBnTT10-3-3*	A03	EM46ME43/419bp–SWUA03-1858-238	63.55	3.30	−0.33	9.98
*qBnTT10-16-4*	C06	EM04ME22/450bp–EM18ME41/330bp	93.30	4.57	−0.48	14.62
*qBnTT12-3-1*	A03	BnGMS417(A03)/190–H061P05-3(A03)/1200	163.15	3.62	−0.24	6.41
*qBnTT12-9-2*	A09	B010D15-4(A09)/940–H112B21-1(A09)/990	74.04	5.55	−0.84	28.01
*qBnTT12-16-3*	C06	SWUC363(C06)–BRMS-195/250bp	61.08	3.65	−0.24	6.47
*qBnTT12-18-4*	C08	SWUC527(C08)–SWUC421(C03/C08)	10.01	8.15	−0.50	28.28
*qBnTT15-1-1*	A01	BRMS-317/400(r1)–BRMS-056/400(r1)	97.64	2.76	0.10	8.86
*qBnTT15-7-2*	A07	EM32ME52/120bp–EM22ME55/190bp	53.50	3.45	0.12	12.95
*qBnTT15-17-3*	C07	SWUC001(C07)–SWUC07_1799	37.35	2.60	−0.12	11.40
*qBnTT18-5-1*	A05	EM29ME03/190bp–BnGMS91a(A05)	79.25	3.50	0.29	8.66
*qBnTT18-9-2*	A09	SWUA09-55-15–SWUA09-2	21.54	6.92	−0.77	28.21
*qBnTT18-9-3*	A09	KS50470(R09)/350–KS30880(A09)/300	73.94	16.84	−1.33	44.48
*qBnTT18-11-4*	C01	SWUC01_1239–SWUC099b(C01)	33.29	2.57	0.26	6.21
*qBnTT18-18-5*	C08	SWUC527(C08)–SWUC421(C03/C08)	10.01	13.24	0.65	40.54
*qBnTT19-6-1*	A06	EM43ME12/200bp–EM58ME09/320bp	58.15	3.36	−0.39	8.49
*qBnTT19-8-2*	A08	EM28ME21/570bp–EM63ME07/1200bp	73.40	2.77	−0.34	6.05
*qBnTT19-9-3*	A09	SWUA09-55-15–SWUA09-2	21.54	17.59	−1.75	53.11
*qBnTT19-14-4*	C04	EM42ME14/140bp–EM04ME14/90bp	131.20	3.74	−0.48	12.36
*qBnTT19-18-5*	C08	SWUC421(C03/C08)–EM21ME40/700bp	21.37	2.86	0.70	23.54
*qBnBAN-3-1*	A03	SWUA03-1871-276–SWUA03-1847-278	68.45	5.72	−0.68	15.54
*qBnBAN-9-2*	A09	SWUA09-63-26–B082F21-2(R09)/300	34.17	17.86	−2.56	52.70
*qBnBAN-9-3*	A09	B055B21-5(A09)/1000–KS30880(A09)/300	74.04	11.94	−1.35	29.64
*qBnBAN-14-4*	C04	EM12ME19/180bp–EM45ME40/390bp	4.50	3.91	−0.50	12.64
*qBnAHA10-1-1*	A01	BRMS-098/180(r1)–EM33ME24/80bp	112.33	3.34	−0.18	8.21
*qBnAHA10-7-2*	A07	Ra2-A01(7)–EM45ME09/300bp	108.56	6.57	−0.26	17.25
*qBnAHA10-15-3*	C05	SWUC090(C05)–SWUC088a(C05)	95.34	2.51	−0.15	6.03
*qBnTT1-7-1*	A07	CB10439(7/11)–SWUC142(C08/C09)	107.79	3.70	0.30	11.35
*qBnTT1-9-2*	A09	SWUA09-17–SWUA09-63-9	36.77	5.21	−0.43	18.40
*qBnTT1-13-3*	C03	SWUC307(C03)–SWUC111(C03)	159.05	6.02	0.95	16.26
*qBnTT1-16-4*	C06	SWUC025(C06)–FITO-095/290	56.74	2.23	0.27	9.26
*qBnTT1-18-5*	C08	SWUC527(C08)–SWUC421(C03/C08)	10.01	6.30	−0.46	26.76
*qBnTT2-1-1*	A01	SWUA01-1064-278a–FITO-101/280	95.24	17.54	−4.74	49.63
*qBnTT2-7-2*	A07	S350/600bp–EM11ME20/190bp	130.60	3.02	−0.44	8.67
*qBnTT2-13-3*	C03	SWUC402(C03)–SWUC558(C03)	157.68	11.48	−1.93	27.04
*qBnTT8-5-1*	A05	CN53/400–EM47ME53/160bp	39.27	4.68	−0.21	11.26
*qBnTT8-9-2*	A09	B010D15-4(A09)/940–H112B21-1(A09)/990	74.04	2.58	−0.16	6.04
*qBnTT8-11-3*	C01	SWUC01_1527–Ol10-A11(11)	38.04	6.21	−0.25	15.65
*qBnTT8-18-4*	C08	SWUC527(C08)–SWUC421(C03/C08)	10.01	7.57	−0.32	24.57
*qBnTT16-6-1*	A06	EM28ME21/450bp–S362/650bp	71.26	3.18	0.19	9.55
*qBnTT16-9-2*	A09	SWUA09-55-5–SWUA09-53	50.06	3.52	−0.19	10.85
*qBnTT16-11-3*	C01	EM03ME17/300bp–CB10536b(1/11)	81.90	5.09	−0.66	17.03
*qBnTTG1-5-1*	A05	SWUA05-520-179–BRMS-057/110(r5)	58.61	3.49	−0.13	8.83
*qBnTTG1-9-2*	A09	KBrB019I24.2/450–KBrB019I24.4/450	46.75	15.64	−0.54	49.20
*qBnTTG1-11-3*	C01	FITO-016/250–EM29ME10/190bp	69.92	4.43	0.24	12.25
*qBnTTG2-6-1*	A06	niab_ssr037(A06)/350–SWUA06-687-153	80.87	6.55	−0.21	22.59
*qBnTTG2-9-2*	A09	SWUA09-63-23A–SWUA09-2	25.12	2.01	−0.19	5.25
*qBnTTG2-11-3*	C01	CB10258(1/11)–SWUC01_100	101.59	2.94	−0.42	17.06
*qBnTTG2-13-4*	C03	EM54ME29/150bp–EM34ME42/400bp	90.29	3.45	0.27	10.37

a *Markers in an eQTL region that flank the peak of the LOD scan*.

b *Additive effects: a positive value (+) indicates that the allele was derived from the GH06 parent, while a negative value (−) indicates that the allele came from the ZY821 parent*.

c *Phenotypic variation explained by eQTL (percentage)*.

### Analysis of flanking sequences of *trans*-eQTLs

To determine whether the eQTLs were *cis* or *trans*, the chromosomal distribution of all characterized *tt* genes on *B. napus, B. rapa*, and *B. oleracea* were obtained based on BLASTN analysis. We found that only 5 of 18 genes were mapped to a similar chromosomal location as their eQTLs, implying that five eQTLs (i.e., *qBnTT1-16-4, qBnTT3-9-2, qBnTT4-13-3, qBnTT5-9-2*, and *qBnTT18-11-4*) were *cis*-eQTLs, whereas the remaining eQTLs were *trans*-eQTLs that controlled the expression of target genes at distant locations.

**Figure 4:**
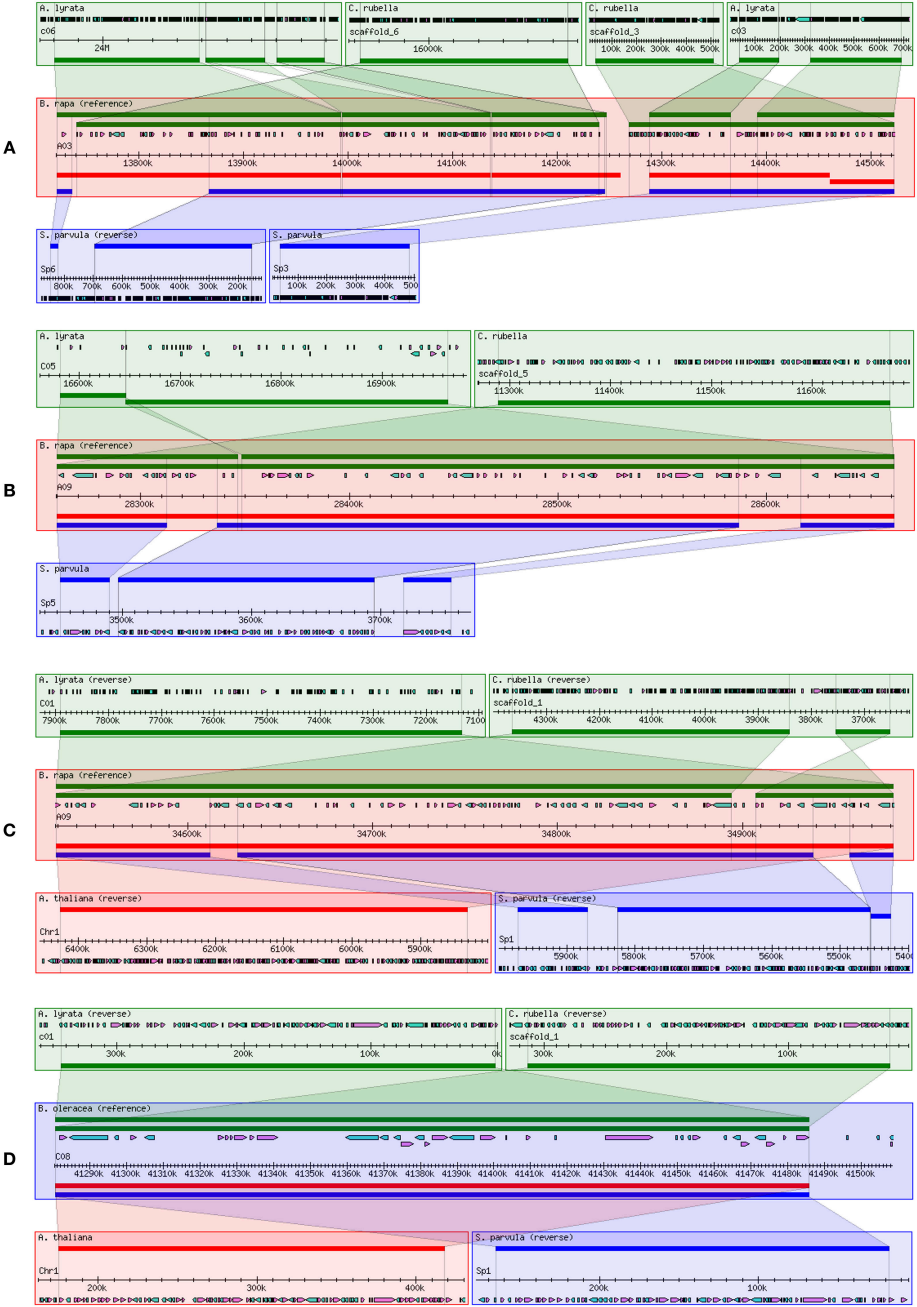
**Comparison of collinearity of ***trans***-eQTL flanking sequences between two ***Brassica*** species and their relatives. (A)** eQTL hotspot on chromosome A03 of *B. napus*; **(B)** lower eQTL hotspot on chromosome A09 of *B. napus*; **(C)** upper eQTL hotspot on chromosome A09 of *B. napus*; and **(D)** eQTL hotspot on chromosome C08 of *B. napus*. Collinearity was analyzed and visualized using the *Brassica* Synteny Blocks tool in the BRAD database (http://brassicadb.org/cgi-bin/gbrowse_syn/brassica/).

Twenty-eight eQTLs for 12 genes were identified in four eQTL hotspots that almost were *trans*-eQTLs. We thus assumed that four eQTL hotspots might include important regulators of flavonoid biosynthesis in *B. napus*. Hence, the 200-kb flanking sequences of core markers of each *trans*-eQTL in *B. napus* were extracted and annotated based on the *B. napus* “Darmor-Bzh” reference genome (http://www.genoscope.cns.fr/brassicanapus/) (Supplementary Table S5). The collinearity of these *trans*-eQTL flanking sequences among *Brassica* species was also determined from *Brassica* Synteny Blocks in the BRAD database (http://brassicadb.org/brad/viewsyntenic.php) (Figure 4). The flanking sequence of the eQTL hotspot on chromosome A03 of *B. rapa* displayed collinearity with chromosome 4 of *A. thaliana* and chromosome C06 of *A. lyrata* (Figure 4A), while the two hotspots on chromosome A09 of *B. rapa* shared synteny with chromosome C05 of *A. lyrata* and chromosome 1 of *A. thaliana*, respectively (Figures 4B,C). In addition, the flanking sequence of the hotspot on chromosome C08 of *B. oleracea* also showed synteny with chromosome 1 of *A. thaliana* (Figure 4D). Hence, we can identify the potential candidate genes related to in the *trans*-eQTL regions by analyzing the syntenic relationships among them and conducting a comparative genomics analysis.

The candidate genes in the 200-kb of nucleotide sequence flanking the four *trans*-eQTL hotspots were annotated by BLASTN analysis. Because each hotspot contained 6 to 8 *trans*-eQTLs (Figure 2), we inferred that the major candidate gene responsible for downstream expression variation was an upstream regulatory gene that encodes a transcription factor. The most interesting hotspot in our study was the lower hotspot on chromosome A09. A total of seven transcription factors were identified in this region (Supplementary Table S5), two of which belong to the flavonoid biosynthesis-related MYB transcription factor family, including *MYB51* (*BnaA09g44500D*, positive regulator of indolic glucosinolate production) and *MYB52* (*BnaA09g44780D*, positive regulator of cell wall thickening). Associated with the *trans*-eQTL hotspots on chromosomes A03 and C08, and the upper *trans*-eQTL hotspot on chromosome A09, we identified 5, 1, and 10 transcription factor genes, respectively (Supplementary Table S5). Among these genes, those encoding *bZIP25* (*BnaA03g18190D*, positive regulator of seed maturation), *MYC1* (*BnaA09g51900D*, positive regulator of epidermal cell differentiation), and transcription factors of unknown function could be regarded as candidate genes involved in flavonol biosynthesis.

## Discussion

### Genetic map construction and alignment

Genetic maps offer a powerful approach for analyzing the structural and functional evolution of crop plants and for detecting QTLs that can be used for marker-assisted breeding programs. Using different populations, many genetic linkage maps have been constructed in *B. napus* based on different markers (Landry et al., 1991; Ferreira et al., 1994; Foisset et al., 1995; Uzunova et al., 1995; Lombard and Delourme, 2001; Xu et al., 2001; Zhao and Meng, 2003; Liu et al., 2005; Piquemal et al., 2005; Qiu et al., 2006; Fu et al., 2007; Radoev et al., 2008; Cheng et al., 2009; Kim et al., 2009). Moreover, many traits of agronomic importance in *B. napus*, such as seed coat color, oil content, and seed yield, are quantitative with complex genetic bases. Recently, a high-density linkage map was constructed using the *Brassica* 60 K Infinium BeadChip Array (Zou et al., 2012; Delourme et al., 2013; Liu et al., 2013; Zhang et al., 2014; Wang et al., 2015). Genome-specific SSR markers have been widely used for genetic mapping, association mapping, comparative mapping, QTL analysis, and marker-assisted selection (Li et al., 2011). Therefore, we constructed a high-density genetic linkage map using four different kinds of markers, and a total 1087 polymorphic loci (464 for SSR, 97 for RAPD, 451 for SRAP, and 75 for IBP) were mapped to 19 linkage groups, covering 2775 cM of the *B. napus* genome with an average distance between two adjacent markers of 2.55 cM. Furthermore, 184 loci were mapped to chromosome A09 with an average distance between adjacent markers of 0.83 cM, indicating that this approach could be used to identify candidate genes for seed coat color, oil content, and other important agronomic traits on chromosome A09 in *B. napus*. Although 13 interval gaps (adjacent markers > 15 cM) were present on 10 different linkage groups (Table 3, Figure 2), the high-density genetic linkage map constructed in this research could be helpful for fine-mapping and marker-assisted selection (MAS) of many important traits of oilseed rape.

Additionally, Brassica is an ideal genus for studying genome evolution and diversification, because it includes both diploid (*B. rapa*, A = 10; *B. nigra*, B = 8 and *B. oleracea*, C = 9) and allotetraploid (*B. juncea*, AB = 18; *B. napus*, AC = 19 and *B. carinata*, BC = 17) species. Moreover, Brassica and Arabidopsis diverged from a common ancestor approximately 14–20 million years ago (Yang et al., 1999), and the genome of *Brassica* species underwent polyploidization, accompanied by gene deletion and rearrangements (Cavell et al., 1998; Lagercrantz, 1998; Ryder et al., 2001; Babula et al., 2003; Lukens et al., 2003). Therefore, many comparative mapping studies have unraveled the extensive genome homology and microsynteny between the A, B, and C genomes of *Brassica* species and between *Brassica* species and *A. thaliana* (Parkin et al., 2005; Jiang et al., 2011; Wang et al., 2011; Yang et al., 2016). Here, we identified a total of 531 pairs of sequence-informative markers and found that these markers mapped on all 19 linkage groups (Figure 2). Moreover, 237/259 published markers were detected and their positions in the linkage map were found to be in good agreement with the aforementioned genetic maps. The linkage map included 196 specific markers that were newly developed from the *B. rapa* and *B. oleracea* genome (Supplementary Table S3, Figure 2). In addition, 370 of 531 markers were exactly anchored to the corresponding genomes of *Brassica* and *Arabidopsis* through BLASTN analysis, 349 of which were mapped to one locus, 20 to two loci, and 1 to three loci (Supplementary Table S3). Moreover, there was strong collinearity among *B. napus, B. rapa*, and *Arabidopsis*, but the markers were sometimes assigned to different genome linkage groups and the relative physical position of markers was inconsistent (Supplementary Table S3, Supplementary Figures S1, S2). There are two possible explanations for these observations. Firstly, the differences of markers may be inaccuracies in allocations of the RIL population, which could disturb the Mendelian segregation and chromosome abnormalities during map construction. Secondly, extensive segmental duplication and rearrangements are known to have occurred during the polyploidization process of Brassica (Teutonico and Osborn, 1994; Parkin et al., 2005; Panjabi et al., 2008; Yang et al., 2016). Therefore, our results provide insight into the differences in genome structure and gene evolution among *Brassica* species and *A. thaliana*, and can be used to generate an effective MAS strategy that can be used to develop lines with improved agronomic traits.

### Association of flavonoid biosynthesis pathway genes in *B. napus*

Flavonoids are secondary metabolites that are extensively distributed in the plant kingdom, with essential roles in protecting plants against UV radiation, drought, and cold stress, and in color formation in fruits and flowers (Winkel-Shirley, 2002). In *Arabidopsis thaliana*, the flavonoid biosynthesis pathway has been characterized mainly using different *tt* mutants, which have transparent and colorless testa (seed coats) (Holton and Cornish, 1995; Devic et al., 1999; Wan et al., 2002; Xie et al., 2003; Baudry et al., 2006; Lepiniec et al., 2006; Routaboul et al., 2006; Cheng, 2013; Saito et al., 2013). The present study showed that *TT10* and *AHA10* were involved in seed color formation of rapeseed, but these genes have yet to be successfully used in rapeseed breeding programs (Fu et al., 2007; Stein et al., 2013; Zhang et al., 2013). The flavonoid biosynthesis pathways of Brassica species are much more complex than those of *A. thaliana* (Supplementary Figure S3); in addition to consisting of more synthesis-related genes, this pathway is also involved in multi-loci interactions, which have been shown to be involved in the formation of seed coat color in *B*. *napus* (Theander et al., 1977; Marles and Gruber, 2004; Akhov et al., 2009; Qu et al., 2013), and dozens of homologous genes in the *B*. *napus* flavonoid biosynthesis pathway have been cloned and characterized (Wei et al., 2007; Xu et al., 2007; Ni et al., 2008; Akhov et al., 2009; Auger et al., 2009; Chai et al., 2009; Lu et al., 2009; Chen et al., 2013). Prior to this study, no comprehensive analysis of the flavonoid biosynthesis pathway had been conducted in *B. napus*. Our previous results showed that the absence of pigment synthesis in the yellow-seeded line of *B. napus* involves the down-regulation, but not complete inactivation, of several key genes in the flavonoid pathway (Qu et al., 2013). In this study, our correlation analysis showed that the expression levels of any two structural genes (*BnTT3, BnTT4, BnTT5, BnTT6, BnTT12, BnTT18*, and *BnAHA10*) and regulatory genes (*BnTT1, BnTT8*, and *BnTTG1*) had a significant positive correlation (*R*^2^ < 0.01), but a significant negative correlation was observed between *BnTT7* and *BnTT10* or *BnBAN* and *BnTT19*, respectively (Table 1), in accordance with our previous research (Qu et al., 2013). Furthermore, we performed a genome-wide comparative analysis between *A. thaliana* and *Brassica* species. The orthologous genes identified in this analysis might be associated with the fact that they have a common evolutionary ancestor (Figure 3). Therefore, our results will be helpful for determining the relationship between and functionalization of these flavonoid biosynthesis genes, and it is necessary to identify the upstream regulatory network that modulates the flavonoid biosynthesis pathway in *B. napus*.

**Figure 5:**
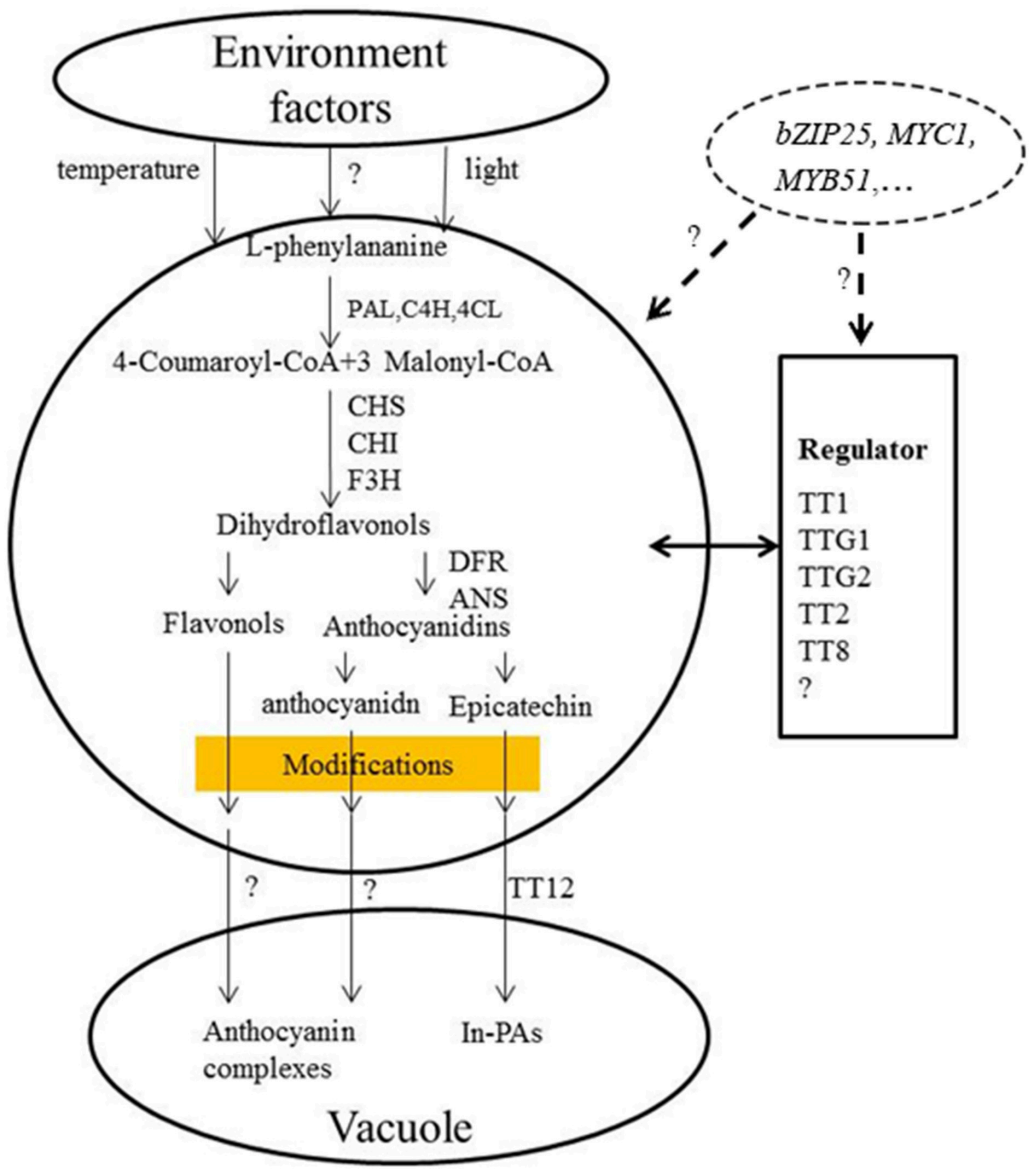
**Proposed model for the flavonoid biosynthesis pathway underlying seed coat color in ***B. napus*****. *BnPAL*, l-phenylalanine ammonialyase; *BnC4H*, cinnamate 4-hydroxylase; *Bn4CL*, 4-coumarate:CoA ligase; *BnCHS*, chalcone synthase; *BnCHI*, chalcone isomerase; *BnF3H*, flavanone-hydroxylase; *BnDFR*, dihydroflavonol reductase; *BnANS*, anthocyanidin synthase; *BnANR*, anthocyanidin reductase (Qu et al., 2013).

Studies have shown that eQTLs provide a basis for deciphering the regulatory networks of genes that modulate pathways in different plants (Brem et al., 2002; Schadt et al., 2003; Morley et al., 2004; Civelek and Lusis, 2014). In this study, the expression profile of each gene in the RILs was used as a quantitative trait, and the eQTLs of these genes was detected by QTL mapping using WinQTL Cartographer 2.5 software. In total, 72 eQTLs were detected and distributed on 15 different linkage groups, with 3 to 5 eQTLs per gene (Table 2, Figure 2). Importantly, 28 eQTLs associated with 12 genes in 4 eQTL hotspots were identified and distributed on chromosomes A03, A09, and C08, respectively. Moreover, the positive alleles of 23 eQTLs associated with seven genes were derived from the male parent ZY821 (Table 3), explaining 4.11–52.70% of the phenotypic variance. These results showed that the eQTLs are distributed in clusters on chromosomes, and help to identify the common regulator gene in major eQTL regions. Based on BLASTN analysis, however, most of the eQTLs were found to be *trans*-eQTLs, controlling the expression of distant target genes. Moreover, 6–8 *trans*-eQTLs were detected on the four hotspots (Table 3, Figure 2), suggesting that these *trans*-eQTLs had essential roles in the flavonoid biosynthesis pathway. Based on the *B. napus* reference genome, some transcription factors related to flavonoid biosynthesis were identified in the eQTL hotspot regions (Supplementary Table S5) associated with members of the R2R3-type MYB gene family (e.g., *MYB51* and *MYB52*), which act as regulators of different pathways (Chen et al., 2006). In addition, one basic leucine zipper (bZIP) transcription factor (*bZIP25*) that interacted with *bZIP10* and *ABI3* to regulate their seed-specific expression during seed maturation (Lara et al., 2003), and one basic Helix-Loop-Helix (bHLH) transcription factor, *MYC1*, that controlled flavonoid biosynthesis and epidermal cell fate (Hichri et al., 2010; Pesch et al., 2013), were also identified. Findings in *A. thaliana* have confirmed that the MYB and bHLH proteins were involved in regulating the flavonoid biosynthesis pathways (Baudry et al., 2006; Dubos et al., 2008; Kitamura et al., 2010; Stracke et al., 2010). Moreover, MYB transcription factors interact with bHLH proteins to regulate flavonoid biosynthesis in plant species (Koes et al., 2005; Quattrocchio et al., 2006). In addition, *TT2* (R2R3-MYB), *TT8* (bHLH), and *TTG1* (WDR) modulate proteins, including DFR, LDOX, BAN, and TT12, thereby affecting PA production, and form a complex called MBW (MYB-bHLH-WD40) in the flavonoid pathway (Baudry et al., 2004, 2006; Lepiniec et al., 2006). Previous studies have proposed *TTG1, TT8, TT10, TT12*, and *AHA10* as candidate genes involved in seed coat color formation in *Brassica* species (Xie et al., 2003; Fu et al., 2007; Chai et al., 2009; Li et al., 2012; Stein et al., 2013; Zhang et al., 2013; Padmaja et al., 2014). Therefore, we predict that the candidate genes *bZIP25, MYC1*, and *MYB51* are involved in the flavonoid biosynthesis pathway through different regulator networks in rapeseed (Figure 5). These results provide useful information for deciphering the upstream regulatory network of the flavonoid gene families and for characterizing transcription factors of unknown function. The genes identified in our study as being involved in flavonol biosynthesis provide insight into the molecular and biochemical mechanism underlying seed coat development in *Brassicaceae*, and might ultimately elucidate the regulatory network underlying seed coat color formation.

## Author contributions

CQ, FF, and KL conceived of the study and drafted the manuscript. HZ and KZ performed the data mining and bioinformatics analysis. JY and LL carried out gene expression analysis and map construction. RW and XX acquired the reagents and conducted the field experiments. KL and JL interpreted the data and revised the manuscript. All authors read and approved of the final manuscript.

### Conflict of interest statement

The authors declare that the research was conducted in the absence of any commercial or financial relationships that could be construed as a potential conflict of interest.
