# Supplementary material for: Molecular Mapping and QTL for Expression Profiles of Flavonoid Genes in Brassica napus
Source: Front Plant Sci. 2016 Nov 9;7:1691. doi: 10.3389/fpls.2016.01691 (PMC5102069; doi:10.3389/fpls.2016.01691)
Supplement: Supplementary file 1 [file Table1.DOCX]

**Supplementary Table S1** Primer sequences of flavonoid genes and a housekeeping gene (*BnACT7* and *BnUBC21*) used for qRT-PCR

| Target gene | Forward sequence (5'- 3') | Reverse sequence (5'- 3') |
| --- | --- | --- |
| *BnTT3* | AGACCGTGTGCGTAACCGGC | AGGATCGCGAACAGTGGCACG |
| *BnTT4* | GACTACTACTTCCGCATCACCAACAG | GCCTAGCTTAGGGACTTCAACAACC |
| *BnTT5* | CTTCCTCGGTGGCGCAGGTG | ACACAGTTCTCCGTTACTTTCTCTGA |
| *BnTT6* | TGGGTGAAAGTGACGGAGGAGT | TGGTTCCAGGGTCAGTGTGACG |
| *BnTT7* | GCCATAGCCCGTGACCCGGA | GCTTCTCCGGCGTAACTCCTCC |
| *BnTT10* | GCGACTGTGCCAAGAAACGGT | CCCCACGTGAGATGTCTATCAAAGTG |
| *BnTT12* | GCTCCACAGAGACATACGAGCCG | ACGGTGACGAAGCTGAGCATGTA |
| *BnTT15* | ACAAAATGACGGGACAGTGGAAGTT | GGCTGCACATCGCCTCGAGTT |
| *BnTT18* | GGCTTAGAGCCTGACCGTCTAGAGAA | TGAGCTTCCACGCCAAGTGCT |
| *BnTT19* | ACATCTTCTTCGTCAGCCATTTGGTCA | GGTCCACGATGGCTCGGTGC |
| *BnBAN* | GGACTTGTGATGACCGAAGAAAACTG | ATGTAGCGACCAGAAGCTGTTTCTTT |
| *BnAHA10* | ACCCATTGCCATGCCCACTGT | GCTCGGCCTGCAAGCAACAA |
| *BnTT1* | TCGCTACAACAATCTTCAGATGCACA | TCCTGCACCCTTCAACGCAGC |
| *BnTT2* | AGCTGGTCTCAAGAGGTGTGGCA | AGCCTCCCAGCTATCAACGACC |
| *BnTT8* | GGCTGAAGAGGCTGCGTCGG | GTGCTGTGCAAGCCCTCGCT |
| *BnTT16* | TGCTCACATCGGTCTCATCGTCT | GCTCGTGTGGAGGAATGGAGGC |
| *BnTTG1* | TCCTCCGGCGACTTCCTCCG | GCTGCGTCTCCACCACGGAC |
| *BnTTG2* | AAACCTAAAGCAAAGCTTGTCTCCCA | ACTTCCTTTGACTTGCTTCTGTCCGT |
| *BnACTIN7* | TGGGTTTGCTGGTGACGAT | TGCCTAGGACGACCAACAATACT |
| *BnUBC21* | CCTCTGCAGCCTCCTCAAGT | CATATCTCCCCTGTCTTGAAATGC |
