# Supplementary material for: Molecular Mapping and QTL for Expression Profiles of Flavonoid Genes in Brassica napus
Source: Front Plant Sci. 2016 Nov 9;7:1691. doi: 10.3389/fpls.2016.01691 (PMC5102069; doi:10.3389/fpls.2016.01691)
Supplement: Supplementary file 2 [file Table2.DOCX]

**Supplementary Table S2** Analysis of the Relative expression level of flavonoid biosynthesis genes for the parents and RILs of *B. napus*

| Gene Name | Parents | | RIL population | | | | | |
| --- | --- | --- | --- | --- | --- | --- | --- | --- |
|  | Zhongyou821 | GH06 Mean | Mean±SE | Max | Min | Stand var. | Skewness | Kurtosis |
| *BnTT3* | CK | 0.20^**^ | 0.83 ± 0.05 | 2.43 | 0.14 | 0.48 | 1.00 | 0.98 |
| *BnTT4* |  | 0.14^**^ | 2.99 ± 0.26 | 8.69 | 0.33 | 2.52 | 0.67 | -1.00 |
| *BnTT5* |  | 0.30^**^ | 1.72 ± 0.09 | 4.35 | 0.37 | 0.96 | 0.79 | -0.23 |
| *BnTT6* |  | 0.16^**^ | 3.15 ± 0.23 | 8.46 | 0.50 | 2.29 | 0.65 | -0.92 |
| *BnTT7* |  | 0.31^**^ | 0.52 ± 0.05 | 1.79 | 0.04 | 0.46 | 1.22 | 0.53 |
| *BnTT10* |  | 0.20^**^ | 1.12 ± 0.05 | 3.03 | 0.02 | 0.57 | 0.70 | 0.69 |
| *BnTT12* |  | 0.21^**^ | 1.40 ± 0.08 | 3.61 | 0.29 | 0.85 | 0.89 | -0.14 |
| *BnTT15* |  | 0.82^*^ | 0.84 ± 0.02 | 1.48 | 0.50 | 0.20 | 0.88 | 1.36 |
| *BnTT18* |  | 0.18^**^ | 2.08 ± 0.14 | 6.68 | 0.45 | 1.40 | 1.09 | 0.48 |
| *BnTT19* |  | 0.17^**^ | 0.80 ± 0.07 | 3.01 | 0.13 | 0.70 | 1.38 | 1.32 |
| *BnBAN* |  | 0.07^**^ | 1.21 ± 0.09 | 4.00 | 0.05 | 0.96 | 0.97 | 0.09 |
| *BnAHA10* |  | 0.46^**^ | 1.63 ± 0.07 | 3.68 | 0.62 | 0.67 | 0.81 | 0.28 |
| *BnTT1* |  | 0.33^**^ | 1.64 ± 0.10 | 4.06 | 0.46 | 0.93 | 0.82 | -0.29 |
| *BnTT2* |  | 0.83^*^ | 0.91 ± 0.04 | 1.95 | 0.28 | 0.35 | 0.62 | 0.26 |
| *BnTT8* |  | 0.50^*^ | 1.45 ± 0.07 | 4.26 | 0.48 | 0.65 | 1.35 | 3.11 |
| *BnTT16* |  | 1.54^*^ | 1.14 ± 0.04 | 2.23 | 0.53 | 0.38 | 1.00 | 0.56 |
| *BnTTG1* |  | 0.42^**^ | 1.38 ± 0.04 | 2.71 | 0.69 | 0.41 | 0.99 | 1.53 |
| *BnTTG2* |  | 0.43^**^ | 0.71 ± 0.03 | 1.56 | 0.21 | 0.31 | 0.93 | 0.40 |

Relative gene expression levels were normalized according to the expression values of the male parent ZY821.^*^, ^**^: was based on Student’s *t*-test: ^*^, *P* < 0.05; and ^**^, *P* < 0.01.
