# Supplementary material for: Molecular Mapping and QTL for Expression Profiles of Flavonoid Genes in Brassica napus
Source: Front Plant Sci. 2016 Nov 9;7:1691. doi: 10.3389/fpls.2016.01691 (PMC5102069; doi:10.3389/fpls.2016.01691)
Supplement: Supplementary file 5 [file Table5.DOCX]

**Supplementary Table S5** Annotation of genes in the flanking sequences of four *trans*-eQTL hotspots

| Hotspot | Genes ID | AGI NO. | Description |
| --- | --- | --- | --- |
| A03 | BnaA03g00590D | AT3G53500 | RSZ32 |
|  | BnaA03g03460D | AT2G37410 | Translocase inner membrane subunit 17-2 (TIM17-2) |
|  | BnaA03g09420D | AT1G14800 | Nucleic acid-binding, OB-fold-like protein |
|  | BnaA03g14360D | AT3G01610 | Cell division cycle 48C (CDC48C) |
|  | BnaA03g14510D | AT2G31530 | EMBRYO DEFECTIVE 2289 (EMB2289) |
|  | BnaA03g16790D | AT3G53000 | Phloem protein 2-A15 (PP2-A15) |
|  | BnaA03g16910D | AT2G36985 | ROTUNDIFOLIA4 (ROT4) |
|  | BnaA03g17070D | AT3G53450 | Putative lysine decarboxylase family protein |
|  | BnaA03g17200D | AT2G37410 | Translocase inner membrane subunit 17-2 (TIM17-2) |
|  | BnaA03g17210D | AT2G37430 | C2H2 and C2HC zinc fingers superfamily protein |
|  | BnaA03g17320D | AT3G53750 | Actin 3 (ACT3) |
|  | BnaA03g17760D | AT3G53900 | Uracil phosphoribosyltransferase (UPP) |
|  | BnaA03g18190D | AT3G54620 | Basic leucine zipper 25 (BZIP25) |
|  | BnaA03g18250D | AT3G54700 | Phosphate transporter 1 |
|  | BnaA03g21210D | AT4G00430 | TRANSMEMBRANE PROTEIN C (TMP-C) |
|  | BnaA03g26580D | AT4G01650 | Polyketide cyclase / dehydrase and lipid transport protein |
|  | BnaA03g26590D | AT4G01670 | Unknown protein |
|  | BnaA03g26600D | AT4G01575 | Serine protease inhibitor, Kazal-type family protein |
|  | BnaA03g26620D | AT4G01450 | Nodulin MtN21 /EamA-like transporter family protein |
|  | BnaA03g26660D | AT4G01450 | Nodulin MtN21 /EamA-like transporter family protein |
|  | BnaA03g26670D | AT4G01410 | Late embryogenesis abundant (LEA) hydroxyproline-rich glycoprotein family |
|  | BnaA03g26680D | AT4G01400 | FUNCTIONS IN: molecular_function unknown |
|  | BnaA03g26700D | AT4G00810 | 60S acidic ribosomal protein family |
|  | BnaA03g26720D | AT4G00830 | RNA-binding (RRM/RBD/RNP motifs) family protein |
|  | BnaA03g26730D | AT4G00840 | DHHC-type zinc finger family protein |
|  | BnaA03g26740D | AT4G00910 | Aluminium-activated malate transporter family protein |
|  | BnaA03g26750D | AT4G00990 | Transcription factor jumonji (jmjC) domain-containing protein |
|  | BnaA03g26760D | AT4G00990 | Transcription factor jumonji (jmjC) domain-containing protein |
|  | BnaA03g26780D | AT4G01010 | Cyclic nucleotide-gated channel 13 (CNGC13) |
|  | BnaA03g26790D | AT4G01026 | PYR1-like 7 (PYL7) |
|  | BnaA03g26800D | AT4G01040 | Glycosyl hydrolase superfamily protein |
|  | BnaA03g26810D | AT4G01050 | Thylakoid rhodanese-like (TROL) |
|  | BnaA03g26820D | AT4G01060 | CAPRICE-like MYB3 (CPL3) |
|  | BnaA03g26840D | AT4G01100 | Adenine nucleotide transporter 1 (ADNT1) |
|  | BnaA03g26850D | AT4G01110 | Unknown protein |
|  | BnaA03g26860D | AT4G01130 | GDSL-like Lipase/Acylhydrolase superfamily protein |
|  | BnaA03g26870D | AT4G01150 | Unknown protein |
|  | BnaA03g26890D | AT4G01210 | Glycosyl transferase family 1 protein |
|  | BnaA03g26910D | AT4G01270 | RING/U-box superfamily protein |
|  | BnaA03g26920D | AT4G01290 | Unknown protein |
|  | BnaA03g26930D | AT4G01310 | Ribosomal L5P family protein |
|  | BnaA03g26940D | AT4G01320 | ATSTE24 |
|  | BnaA03g26950D | AT4G01370 | MAP kinase 4 (MPK4) |
|  | BnaA03g26960D | AT4G00770 | Unknown protein |
|  | BnaA03g26970D | AT4G00755 | F-box family protein |
|  | BnaA03g26980D | AT4G00752 | UBX domain-containing protein |
|  | BnaA03g26990D | AT4G00740 | S-adenosyl-L-methionine-dependent methyltransferases superfamily protein |
|  | BnaA03g27010D | AT4G00720 | Shaggy-like protein kinase 32 (SK32) |
|  | BnaA03g27020D | AT4G00710 | BR-signaling kinase 3 (BSK3) |
|  | BnaA03g27030D | AT4G00700 | C2 calcium/lipid-binding plant phosphoribosyltransferase family protein |
|  | BnaA03g27040D | AT4G00670 | Remorin family protein |
|  | BnaA03g27050D | AT4G00660 | RNAhelicase-like 8 (RH8) |
|  | BnaA03g27060D | AT4G00585 | Unknown protein |
|  | BnaA03g27070D | AT4G00570 | NAD-dependent malic enzyme 2 (NAD-ME2) |
|  | BnaA03g27080D | AT4G00525 | Unknown protein |
|  | BnaA03g27100D | AT4G00500 | Alpha/beta-Hydrolases superfamily protein |
|  | BnaA03g27110D | AT4G00450 | CENTER CITY (CCT) |
|  | BnaA03g27120D | AT4G00440 | FUNCTIONS IN: molecular_function unknown |
|  | BnaA03g27130D | AT4G00430 | TRANSMEMBRANE PROTEIN C (TMP-C) |
|  | BnaA03g27140D | AT4G00370 | ANTR2 |
|  | BnaA03g27150D | AT4G00355 | Unknown protein |
|  | BnaA03g27170D | AT4G00170 | Plant VAMP (vesicle-associated membrane protein) family protein |
|  | BnaA03g27180D | AT4G00120 | INDEHISCENT (IND) |
|  | BnaA03g27200D | AT3G02000 | ROXY1 |
|  | BnaA03g27210D | AT3G01990 | ACT domain repeat 6 (ACR6) |
|  | BnaA03g27220D | AT3G01980 | NAD(P)-binding Rossmann-fold superfamily protein |
|  | BnaA03g27230D | AT3G01870 | Plant protein of unknown function (DUF946) |
|  | BnaA03g27240D | AT3G01860 | Unknown protein |
|  | BnaA03g27250D | AT3G01830 | Calcium-binding EF-hand family protein |
|  | BnaA03g27260D | AT3G01820 | P-loop containing nucleoside triphosphate hydrolases superfamily protein |
|  | BnaA03g27270D | AT3G01810 | FUNCTIONS IN: molecular_function unknown |
|  | BnaA03g27280D | AT3G01800 | Ribosome recycling factor |
|  | BnaA03g27290D | AT3G01790 | Ribosomal protein L13 family protein |
|  | BnaA03g27300D | AT3G01750 | Ankyrin repeat family protein |
|  | BnaA03g27310D | AT3G01740 | Mitochondrial ribosomal protein L37 |
|  | BnaA03g27320D | AT3G01730 | Unknown protein |
|  | BnaA03g27330D | AT3G01720 | Unknown protein |
|  | BnaA03g27340D | AT3G01710 | TPX2 (targeting protein for Xklp2) protein family |
|  | BnaA03g27350D | AT3G01700 | Arabinogalactan protein 11 (AGP11) |
|  | BnaA03g27360D | AT3G01670 | Unknown protein |
|  | BnaA03g27370D | AT3G01650 | RING domain ligase1 (RGLG1) |
|  | BnaA03g27380D | AT3G01640 | Glucuronokinase G (GLCAK) |
|  | BnaA03g27390D | AT3G01610 | Cell division cycle 48C (CDC48C) |
|  | BnaA03g27400D | AT3G01590 | Galactose mutarotase-like superfamily protein |
|  | BnaA03g30540D | AT2G37120 | S1FA-like DNA-binding protein |
|  | BnaA03g31930D | AT3G11740 | Protein of unknown function (DUF567) |
|  | BnaA03g36170D | AT1G17720 | ATB BETA |
|  | BnaA03g37440D | AT3G54670 | TITAN8 (TTN8) |
|  | BnaA03g37450D | AT3G54660 | Glutathione reductase (GR) |
|  | BnaA03g39000D | AT3G01610 | Cell division cycle 48C (CDC48C) |
|  | BnaA03g39070D | AT1G14800 | Nucleic acid-binding, OB-fold-like protein |
|  | BnaA03g42830D | AT4G01400 | FUNCTIONS IN: molecular_function unknown |
|  | BnaA03g45580D | AT1G14800 | Nucleic acid-binding, OB-fold-like protein |
|  | BnaA03g45950D | AT4G00430 | TRANSMEMBRANE PROTEIN C (TMP-C) |
|  | BnaA03g51360D | AT4G31180 | Class II aminoacyl-tRNA and biotin synthetases superfamily protein |
|  | BnaA03g52160D | AT1G14800 | Nucleic acid-binding, OB-fold-like protein |
|  | BnaA03g53170D | AT3G53310 | AP2/B3-like transcriptional factor family protein |
|  | BnaA03g53790D | AT1G14800 | Nucleic acid-binding, OB-fold-like protein |
|  | BnaA03g54340D | AT4G00585 | Unknown protein |
|  | BnaA03g60630D | AT3G01710 | TPX2 (targeting protein for Xklp2) protein family |
| A09-1 | BnaA09g00060D | AT4G01320 | ATSTE24 |
|  | BnaA09g00070D | AT4G01290 | Unknown protein |
|  | BnaA09g00100D | AT4G01270 | RING/U-box superfamily protein |
|  | BnaA09g00140D | AT4G01210 | Glycosyl transferase family 1 protein |
|  | BnaA09g00150D | AT4G01150 | Unknown protein |
|  | BnaA09g00180D | AT4G01100 | Adenine nucleotide transporter 1 (ADNT1) |
|  | BnaA09g00190D | AT4G01090 | Protein of unknown function (DUF3133) |
|  | BnaA09g00230D | AT4G01060 | CAPRICE-like MYB3 (CPL3) |
|  | BnaA09g00250D | AT4G01050 | Thylakoid rhodanese-like (TROL) |
|  | BnaA09g00290D | AT4G00990 | Transcription factor jumonji (jmjC) domain-containing protein |
|  | BnaA09g00300D | AT4G00990 | Transcription factor jumonji (jmjC) domain-containing protein |
|  | BnaA09g03840D | AT5G27700 | Ribosomal protein S21e |
|  | BnaA09g04070D | AT5G26830 | Threonyl-tRNA synthetase |
|  | BnaA09g11000D | AT1G14800 | Nucleic acid-binding, OB-fold-like protein |
|  | BnaA09g12360D | AT1G63690 | SIGNAL PEPTIDE PEPTIDASE-LIKE 2 (SPPL2) |
|  | BnaA09g12380D | AT1G14800 | Nucleic acid-binding, OB-fold-like protein |
|  | BnaA09g14080D | AT4G00710 | BR-signaling kinase 3 (BSK3) |
|  | BnaA09g14600D | AT4G16130 | Arabinose kinase (ARA1) |
|  | BnaA09g16370D | AT2G35280 | F-box family protein |
|  | BnaA09g16440D | AT3G54700 | Phosphate transporter 1 |
|  | BnaA09g19410D | AT1G14800 | Nucleic acid-binding, OB-fold-like protein |
|  | BnaA09g24920D | AT2G35280 | F-box family protein |
|  | BnaA09g24930D | AT1G14800 | Nucleic acid-binding, OB-fold-like protein |
|  | BnaA09g25690D | AT1G14800 | Nucleic acid-binding, OB-fold-like protein |
|  | BnaA09g25820D | AT1G14800 | Nucleic acid-binding, OB-fold-like protein |
|  | BnaA09g26320D | AT3G54620 | Basic leucine zipper 25 (BZIP25) |
|  | BnaA09g27610D | AT1G14800 | Nucleic acid-binding, OB-fold-like protein |
|  | BnaA09g31180D | AT1G23320 | Tryptophan aminotransferase related 1 (TAR1) |
|  | BnaA09g31200D | AT1G23320 | Tryptophan aminotransferase related 1 (TAR1) |
|  | BnaA09g33320D | AT3G52950 | CBS / octicosapeptide/Phox/Bemp1 (PB1) domains-containing protein |
|  | BnaA09g33330D | AT3G52960 | Thioredoxin superfamily protein |
|  | BnaA09g33350D | AT3G53000 | Phloem protein 2-A15 (PP2-A15) |
|  | BnaA09g33360D | AT3G53010 | Domain of unknown function (DUF303) |
|  | BnaA09g33390D | AT3G53020 | SHORT VALVE1 (STV1) |
|  | BnaA09g33400D | AT3G53030 | Ser/Arg-rich protein kinase 4 (SRPK4) |
|  | BnaA09g33410D | AT3G53150 | UDP-glucosyl transferase 73D1 (UGT73D1) |
|  | BnaA09g33430D | AT3G53150 | UDP-glucosyl transferase 73D1 (UGT73D1) |
|  | BnaA09g33440D | AT3G53160 | UDP-glucosyl transferase 73C7 (UGT73C7) |
|  | BnaA09g33450D | AT3G53170 | Tetratricopeptide repeat (TPR)-like superfamily protein |
|  | BnaA09g33460D | AT3G53180 | Glutamate-ammonia ligases |
|  | BnaA09g33470D | AT3G53190 | Pectin lyase-like superfamily protein |
|  | BnaA09g33480D | AT3G53200 | Myb domain protein 27 (MYB27) |
|  | BnaA09g33490D | AT3G53230 | ATPase, AAA-type, CDC48 protein |
|  | BnaA09g33520D | AT2G36985 | ROTUNDIFOLIA4 (ROT4) |
|  | BnaA09g33550D | AT3G53250 | SAUR-like auxin-responsive protein family |
|  | BnaA09g33560D | AT3G53260 | Phenylalanine ammonia-lyase 2 (PAL2) |
|  | BnaA09g33590D | AT3G53280 | Cytochrome p450 71b5 (CYP71B5) |
|  | BnaA09g33610D | AT3G53300 | Cytochrome P450, family 71, subfamily B, polypeptide 31 (CYP71B31) |
|  | BnaA09g33620D | AT3G53310 | AP2/B3-like transcriptional factor family protein |
|  | BnaA09g33630D | AT3G53320 | Unknown protein |
|  | BnaA09g33640D | AT3G53340 | Nuclear factor Y, subunit B10 (NF-YB10) |
|  | BnaA09g33650D | AT3G53350 | ROP interactive partner 4 (RIP4) |
|  | BnaA09g33660D | AT3G53360 | Tetratricopeptide repeat (TPR)-like superfamily protein |
|  | BnaA09g33670D | AT2G37120 | S1FA-like DNA-binding protein |
|  | BnaA09g33680D | AT3G53390 | Transducin/WD40 repeat-like superfamily protein |
|  | BnaA09g33710D | AT3G53400 | BEST Arabidopsis thaliana protein match is: conserved peptide upstream open reading frame 47 (TAIR:AT5G03190.1) |
|  | BnaA09g33720D | AT3G53420 | Plasma membrane intrinsic protein 2A (PIP2A) |
|  | BnaA09g33730D | AT3G53430 | Ribosomal protein L11 family protein |
|  | BnaA09g33740D | AT3G53440 | Homeodomain-like superfamily protein |
|  | BnaA09g33760D | AT3G53460 | Chloroplast RNA-binding protein 29 (CP29) |
|  | BnaA09g33770D | AT3G53470 | Unknown protein |
|  | BnaA09g33780D | AT3G53500 | RSZ32 |
|  | BnaA09g33810D | AT3G53540 | Unknown protein |
|  | BnaA09g33820D | AT3G53560 | Tetratricopeptide repeat (TPR)-like superfamily protein |
|  | BnaA09g33830D | AT3G53570 | FUS3-complementing gene 1 (FC1) |
|  | BnaA09g33840D | AT3G53580 | Diaminopimelate epimerase family protein |
|  | BnaA09g33850D | AT4G31180 | Class II aminoacyl-tRNA and biotin synthetases superfamily protein |
|  | BnaA09g33860D | AT3G53590 | Leucine-rich repeat protein kinase family protein |
|  | BnaA09g33870D | AT3G53620 | Pyrophosphorylase 4 (PPa4) |
|  | BnaA09g33880D | AT3G53630 | Unknown protein |
|  | BnaA09g33900D | AT3G53670 | Unknown protein |
|  | BnaA09g33910D | AT3G53710 | ARF-GAP domain 6 (AGD6) |
|  | BnaA09g33920D | AT3G53720 | Cation/H+ exchanger 20 (CHX20) |
|  | BnaA09g33940D | AT1G16930 | F-box/RNI-like/FBD-like domains-containing protein |
|  | BnaA09g33970D | AT3G53750 | Actin 3 (ACT3) |
|  | BnaA09g33980D | AT3G53760 | GAMMA-TUBULIN COMPLEX PROTEIN 4 (GCP4) |
|  | BnaA09g34010D | AT3G54090 | Fructokinase-like 1 (FLN1) |
|  | BnaA09g34020D | AT3G54100 | O-fucosyltransferase family protein |
|  | BnaA09g34030D | AT3G54110 | Plant uncoupling mitochondrial protein 1 (PUMP1) |
|  | BnaA09g34040D | AT3G54120 | Reticulon family protein |
|  | BnaA09g34050D | AT4G13460 | SU(VAR)3-9 homolog 9 (SUVH9) |
|  | BnaA09g34060D | AT3G54130 | Josephin family protein |
|  | BnaA09g34070D | AT3G54140 | Peptide transporter 1 (PTR1) |
|  | BnaA09g34080D | AT3G54150 | S-adenosyl-L-methionine-dependent methyltransferases superfamily protein |
|  | BnaA09g34090D | AT3G54170 | FKBP12 interacting protein 37 (FIP37) |
|  | BnaA09g34110D | AT3G54190 | Transducin/WD40 repeat-like superfamily protein |
|  | BnaA09g34120D | AT3G54200 | Late embryogenesis abundant (LEA) hydroxyproline-rich glycoprotein family |
|  | BnaA09g34130D | AT3G54210 | Ribosomal protein L17 family protein |
|  | BnaA09g34140D | AT3G54220 | SCARECROW (SCR) |
|  | BnaA09g34150D | AT3G54230 | Suppressor of abi3-5 (SUA) |
|  | BnaA09g34160D | AT3G54240 | Alpha/beta-Hydrolases superfamily protein |
|  | BnaA09g34170D | AT3G54240 | Alpha/beta-Hydrolases superfamily protein |
|  | BnaA09g34180D | AT3G54250 | GHMP kinase family protein |
|  | BnaA09g34190D | AT3G54270 | Sucrose-6F-phosphate phosphohydrolase family protein |
|  | BnaA09g34200D | AT3G54280 | ROOT GROWTH DEFECTIVE 3 (RGD3) |
|  | BnaA09g34210D | AT3G54280 | ROOT GROWTH DEFECTIVE 3 (RGD3) |
|  | BnaA09g34220D | AT3G54290 | FUNCTIONS IN: molecular_function unknown |
|  | BnaA09g34230D | AT3G54300 | Vesicle-associated membrane protein 727 (VAMP727) |
|  | BnaA09g34240D | AT3G54310 | Unknown protein |
|  | BnaA09g34250D | AT3G54320 | WRINKLED 1 (WRI1) |
|  | BnaA09g34270D | AT3G54360 | Zinc ion binding |
|  | BnaA09g34280D | AT3G54400 | Eukaryotic aspartyl protease family protein |
|  | BnaA09g34290D | AT3G54420 | Homolog of carrot EP3-3 chitinase (EP3) |
|  | BnaA09g34300D | AT3G54430 | SHI-related sequence 6 (SRS6) |
|  | BnaA09g34320D | AT3G54450 | Major facilitator superfamily protein |
|  | BnaA09g34330D | AT3G54480 | SKP1/ASK-interacting protein 5 (SKIP5) |
|  | BnaA09g34360D | AT3G54510 | Early-responsive to dehydration stress protein (ERD4) |
|  | BnaA09g34370D | AT3G54520 | Unknown protein |
|  | BnaA09g34380D | AT3G54510 | Early-responsive to dehydration stress protein (ERD4) |
|  | BnaA09g34390D | AT3G54520 | Unknown protein |
|  | BnaA09g34400D | AT3G54540 | General control non-repressible 4 (GCN4) |
|  | BnaA09g34410D | AT3G54560 | Histone H2A 11 (HTA11) |
|  | BnaA09g34440D | AT3G54610 | Histone acetyltransferase of the GNAT family 1 (HAG1) |
|  | BnaA09g34450D | AT3G54610 | Histone acetyltransferase of the GNAT family 1 (HAG1) |
|  | BnaA09g34460D | AT3G54620 | Basic leucine zipper 25 (BZIP25) |
|  | BnaA09g34470D | AT3G54640 | Tryptophan synthase alpha chain (TSA1) |
|  | BnaA09g34480D | AT3G54650 | FBL17 |
|  | BnaA09g34490D | AT3G54670 | TITAN8 (TTN8) |
|  | BnaA09g34500D | AT3G54680 | Proteophosphoglycan-related |
|  | BnaA09g34510D | AT3G54700 | Phosphate transporter 1 |
|  | BnaA09g34520D | AT3G54720 | ALTERED MERISTEM PROGRAM 1 (AMP1) |
|  | BnaA09g34530D | AT4G00026 | FUNCTIONS IN: molecular_function unknown |
|  | BnaA09g34540D | AT3G54740 | Protein of unknown function, DUF593 |
|  | BnaA09g34550D | AT3G54750 | Unknown protein |
|  | BnaA09g34560D | AT3G54770 | RNA-binding (RRM/RBD/RNP motifs) family protein |
|  | BnaA09g34570D | AT3G54790 | ARM repeat superfamily protein |
|  | BnaA09g34580D | AT3G54800 | Pleckstrin homology (PH) and lipid-binding START domains-containing protein |
|  | BnaA09g34590D | AT3G54810 | BLUE MICROPYLAR END 3 (BME3) |
|  | BnaA09g34600D | AT3G54820 | Plasma membrane intrinsic protein 2 |
|  | BnaA09g34610D | AT3G54826 | Zim17-type zinc finger protein |
|  | BnaA09g34630D | AT2G26110 | Protein of unknown function (DUF761) |
|  | BnaA09g34640D | AT2G39130 | Transmembrane amino acid transporter family protein |
| A09-2 | BnaA09g38290D | AT3G60220 | TOXICOS EN LEVADURA 4 (ATL4) |
|  | BnaA09g39170D | AT4G00430 | TRANSMEMBRANE PROTEIN C (TMP-C) |
|  | BnaA09g39320D | AT3G61600 | POZ/BTB containin G-protein 1 (POB1) |
|  | BnaA09g39370D | AT4G01100 | Adenine nucleotide transporter 1 (ADNT1) |
|  | BnaA09g44500D | AT1G18570 | Myb domain protein 51 (MYB51) |
|  | BnaA09g44520D | AT1G74050 | Ribosomal protein L6 family protein |
|  | BnaA09g44530D | AT1G18530 | EF hand calcium-binding protein family |
|  | BnaA09g44540D | AT1G18520 | Tetraspanin11 (TET11) |
|  | BnaA09g44550D | AT1G18500 | Methylthioalkylmalate synthase-like 4 (MAML-4) |
|  | BnaA09g44560D | AT1G18470 | Transmembrane Fragile-X-F-associated protein |
|  | BnaA09g44570D | AT1G18460 | Alpha/beta-Hydrolases superfamily protein |
|  | BnaA09g44590D | AT1G18390 | Protein kinase superfamily protein |
|  | BnaA09g44600D | AT1G18370 | HINKEL (HIK) |
|  | BnaA09g44610D | AT3G10110 | Maternal effect embryo arrest 67 (MEE67) |
|  | BnaA09g44620D | AT1G18290 | Unknown protein |
|  | BnaA09g44630D | AT1G18280 | Bifunctional inhibitor/lipid-transfer protein/seed storage 2S albumin superfamily protein |
|  | BnaA09g44640D | AT1G18280 | Bifunctional inhibitor/lipid-transfer protein/seed storage 2S albumin superfamily protein |
|  | BnaA09g44650D | AT1G18280 | Bifunctional inhibitor/lipid-transfer protein/seed storage 2S albumin superfamily protein |
|  | BnaA09g44660D | AT5G26830 | Threonyl-tRNA synthetase |
|  | BnaA09g44680D | AT1G18170 | FKBP-like peptidyl-prolyl cis-trans isomerase family protein |
|  | BnaA09g44690D | AT1G18150 | ATMPK8 |
|  | BnaA09g44700D | AT1G18100 | E12A11 |
|  | BnaA09g44710D | AT1G18080 | ATARCA |
|  | BnaA09g44730D | AT1G18040 | Cyclin-dependent kinase D1 |
|  | BnaA09g44740D | AT1G76690 | 12-oxophytodienoate reductase 2 (OPR2) |
|  | BnaA09g44750D | AT1G18010 | Major facilitator superfamily protein |
|  | BnaA09g44760D | AT1G17980 | poly(A) polymerase 1 (PAPS1) |
|  | BnaA09g44770D | AT5G26830 | Threonyl-tRNA synthetase |
|  | BnaA09g44780D | AT1G17950 | Myb domain protein 52 (MYB52) |
|  | BnaA09g44790D | AT1G17950 | Myb domain protein 52 (MYB52) |
|  | BnaA09g44800D | AT1G17880 | Basic transcription factor 3 (BTF3) |
|  | BnaA09g44810D | AT1G17820 | Putative integral membrane protein conserved region (DUF2404) |
|  | BnaA09g44820D | AT1G17810 | Beta-tonoplast intrinsic protein (BETA-TIP) |
|  | BnaA09g44830D | AT1G17790 | DNA-binding bromodomain-containing protein |
|  | BnaA09g44840D | AT1G17720 | ATB BETA |
|  | BnaA09g44860D | AT1G45403 | Membrane protein |
|  | BnaA09g44870D | AT2G04040 | TX1 |
|  | BnaA09g44880D | AT1G17600 | Disease resistance protein (TIR-NBS-LRR class) family |
|  | BnaA09g44890D | AT1G17600 | Disease resistance protein (TIR-NBS-LRR class) family |
|  | BnaA09g44900D | AT1G17610 | Disease resistance protein (TIR-NBS class) |
|  | BnaA09g44910D | AT1G17610 | Disease resistance protein (TIR-NBS class) |
|  | BnaA09g44920D | AT1G17590 | Nuclear factor Y, subunit A8 (NF-YA8) |
|  | BnaA09g44930D | AT1G17610 | Disease resistance protein (TIR-NBS class) |
|  | BnaA09g44940D | AT5G48770 | Disease resistance protein (TIR-NBS-LRR class) family |
|  | BnaA09g44950D | AT1G17580 | Myosin 1 (MYA1) |
|  | BnaA09g44970D | AT1G17520 | Homeodomain-like/winged-helix DNA-binding family protein |
|  | BnaA09g44980D | AT1G17500 | ATPase E1-E2 type family protein / haloacid dehalogenase-like hydrolase family protein |
|  | BnaA09g44990D | AT1G17455 | ELF4-like 4 (ELF4-L4) |
|  | BnaA09g45010D | AT1G17420 | Lipoxygenase 3 (LOX3) |
|  | BnaA09g45020D | AT1G17310 | MADS-box transcription factor family protein |
|  | BnaA09g45030D | AT1G17290 | Alanine aminotransferas (AlaAT1) |
|  | BnaA09g45040D | AT1G17285 | Unknown protein |
|  | BnaA09g45050D | AT1G17280 | Ubiquitin-conjugating enzyme 34 (UBC34) |
|  | BnaA09g45060D | AT1G17200 | Uncharacterised protein family (UPF0497) |
|  | BnaA09g45070D | AT1G17180 | Glutathione S-transferase TAU 25 (GSTU25) |
|  | BnaA09g45080D | AT1G17180 | Glutathione S-transferase TAU 25 (GSTU25) |
|  | BnaA09g45090D | AT1G17180 | Glutathione S-transferase TAU 25 (GSTU25) |
|  | BnaA09g45100D | AT1G17160 | pfkB-like carbohydrate kinase family protein |
|  | BnaA09g45110D | AT1G17160 | pfkB-like carbohydrate kinase family protein |
|  | BnaA09g45130D | AT1G17140 | Interactor of constitutive active rops 1 (ICR1) |
|  | BnaA09g45140D | AT1G17130 | Family of unknown function (DUF572) |
|  | BnaA09g45150D | AT1G17120 | Cationic amino acid transporter 8 (CAT8) |
|  | BnaA09g45160D | AT1G17090 | Unknown protein |
|  | BnaA09g45170D | AT1G17080 | Ribosomal protein L18ae family |
|  | BnaA09g45180D | AT1G17070 | GC-rich sequence DNA-binding factor-like protein with Tuftelin interacting domain |
|  | BnaA09g45190D | AT1G17060 | Cytochrome p450 72c1 (CYP72C1) |
|  | BnaA09g46480D | AT1G12210 | RPS5-like 1 (RFL1) |
|  | BnaA09g46490D | AT1G12210 | RPS5-like 1 (RFL1) |
|  | BnaA09g47220D | AT1G12210 | RPS5-like 1 (RFL1) |
|  | BnaA09g47230D | AT1G12210 | RPS5-like 1 (RFL1) |
|  | BnaA09g51520D | AT4G00810 | 60S acidic ribosomal protein family |
|  | BnaA09g51700D | AT4G00370 | ANTR2 |
|  | BnaA09g51760D | AT4G00755 | F-box family protein |
|  | BnaA09g51770D | AT4G00740 | S-adenosyl-L-methionine-dependent methyltransferases superfamily protein |
|  | BnaA09g51790D | AT4G00720 | Shaggy-like protein kinase 32 (SK32) |
|  | BnaA09g51800D | AT4G00710 | BR-signaling kinase 3 (BSK3) |
|  | BnaA09g51820D | AT4G00660 | RNAhelicase-like 8 (RH8) |
|  | BnaA09g51850D | AT4G00585 | Unknown protein |
|  | BnaA09g51860D | AT4G00570 | NAD-dependent malic enzyme 2 (NAD-ME2) |
|  | BnaA09g51900D | AT4G00480 | ATMYC1 |
|  | BnaA09g51920D | AT4G00460 | RHO guanyl-nucleotide exchange factor 3 (ROPGEF3) |
|  | BnaA09g51930D | AT4G00450 | CENTER CITY (CCT) |
|  | BnaA09g51940D | AT4G00440 | FUNCTIONS IN: molecular_function unknown |
|  | BnaA09g51960D | AT4G00430 | TRANSMEMBRANE PROTEIN C (TMP-C) |
|  | BnaA09g51990D | AT4G01410 | Late embryogenesis abundant (LEA) hydroxyproline-rich glycoprotein family |
|  | BnaA09g52020D | AT4G00810 | 60S acidic ribosomal protein family |
|  | BnaA09g52030D | AT4G00830 | RNA-binding (RRM/RBD/RNP motifs) family protein |
|  | BnaA09g52040D | AT4G00840 | DHHC-type zinc finger family protein |
|  | BnaA09g52080D | AT4G00910 | Aluminium-activated malate transporter family protein |
|  | BnaA09g52150D | AT4G01670 | Unknown protein |
|  | BnaA09g52170D | AT4G01650 | Polyketide cyclase / dehydrase and lipid transport protein |
|  | BnaA09g52200D | AT4G01575 | Serine protease inhibitor, Kazal-type family protein |
|  | BnaA09g52510D | AT5G65380 | MATE efflux family protein |
|  | BnaA09g53830D | AT1G14800 | Nucleic acid-binding, OB-fold-like protein |
|  | BnaA09g54710D | AT3G53540 | Unknown protein |
|  | BnaA09g54720D | AT3G53530 | Chloroplast-targeted copper chaperone protein |
|  | BnaA09g54730D | AT3G53520 | UDP-glucuronic acid decarboxylase 1 (UXS1) |
|  | BnaA09g54750D | AT3G53810 | Concanavalin A-like lectin protein kinase family protein |
|  | BnaA09g54760D | AT3G53800 | Fes1B (Fes1B) |
|  | BnaA09g54890D | AT3G53840 | Protein kinase superfamily protein |
|  | BnaA09g54900D | AT3G53850 | Uncharacterised protein family (UPF0497) |
|  | BnaA09g54910D | AT3G53880 | NAD(P)-linked oxidoreductase superfamily protein |
|  | BnaA09g54920D | AT5G27700 | Ribosomal protein S21e |
|  | BnaA09g54930D | AT3G53900 | Uracil phosphoribosyltransferase (UPP) |
|  | BnaA09g54940D | AT3G53960 | Major facilitator superfamily protein |
|  | BnaA09g54950D | AT3G53970 | Proteasome inhibitor-related |
|  | BnaA09g54960D | AT3G53990 | Adenine nucleotide alpha hydrolases-like superfamily protein |
|  | BnaA09g54970D | AT3G53990 | Adenine nucleotide alpha hydrolases-like superfamily protein |
|  | BnaA09g54980D | AT3G54000 | Unknown protein |
|  | BnaA09g56080D | AT1G18580 | Galacturonosyltransferase 11 (GAUT11) |
|  | BnaA09g56100D | AT1G18620 | Unknown protein |
|  | BnaA09g56110D | AT1G18660 | Zinc finger (C3HC4-type RING finger) family protein |
|  | BnaA09g56120D | AT1G18680 | HNH endonuclease domain-containing protein |
|  | BnaA09g56130D | AT1G18700 | DNAJ heat shock N-terminal domain-containing protein |
