# Supplementary material for: Molecular Mapping and QTL for Expression Profiles of Flavonoid Genes in Brassica napus
Source: Front Plant Sci. 2016 Nov 9;7:1691. doi: 10.3389/fpls.2016.01691 (PMC5102069; doi:10.3389/fpls.2016.01691)
Supplement: Supplementary file 8 [file Image1.PDF]

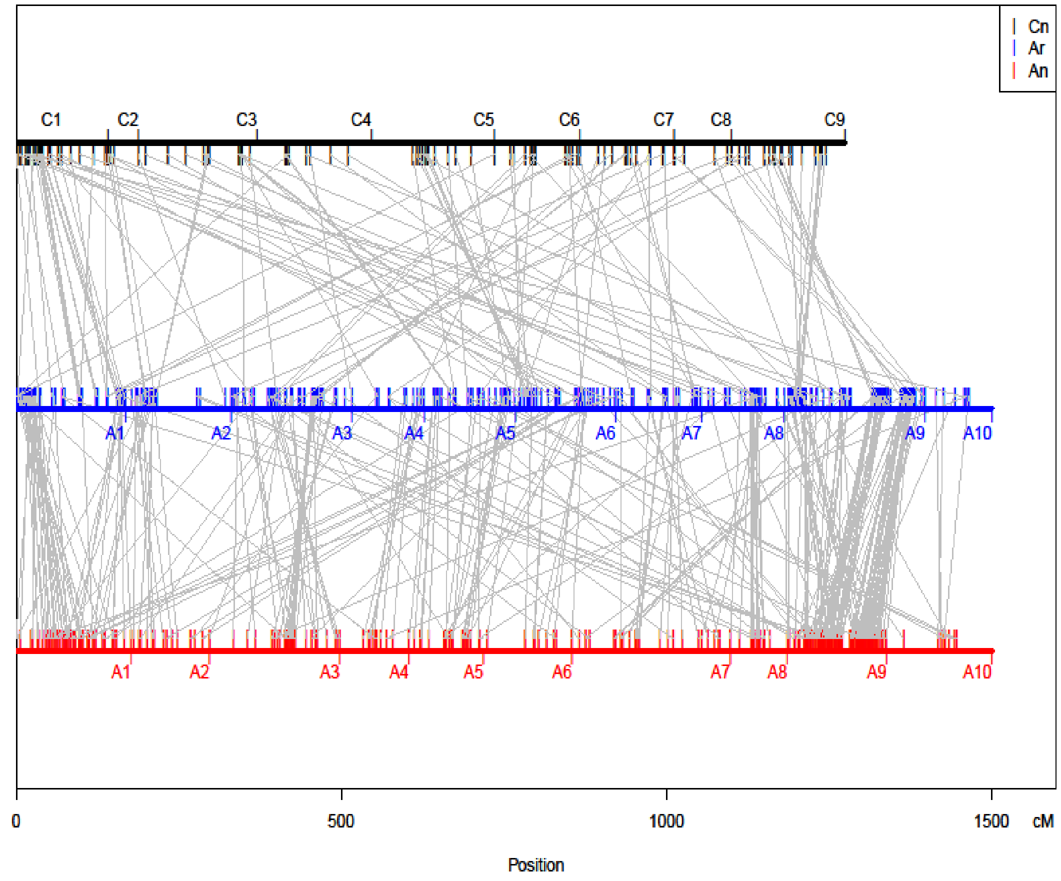

Supplementary Figure S1 Collinearity analysis between the linkage map of *B. napus* and physical map of *B. rapa*. Cn (black), Chromosome C1 to C9 from *B. napus*; Ar (blue), Chromosome A1 to A10 from *B. rapa*; An (red), Chromosome A1 to A10 from *B. napus*. The gray lines indicate the corresponding position of markers in each chromosome.
