# Supplementary material for: Molecular Mapping and QTL for Expression Profiles of Flavonoid Genes in Brassica napus
Source: Front Plant Sci. 2016 Nov 9;7:1691. doi: 10.3389/fpls.2016.01691 (PMC5102069; doi:10.3389/fpls.2016.01691)
Supplement: Supplementary file 10 [file Image3.PDF]

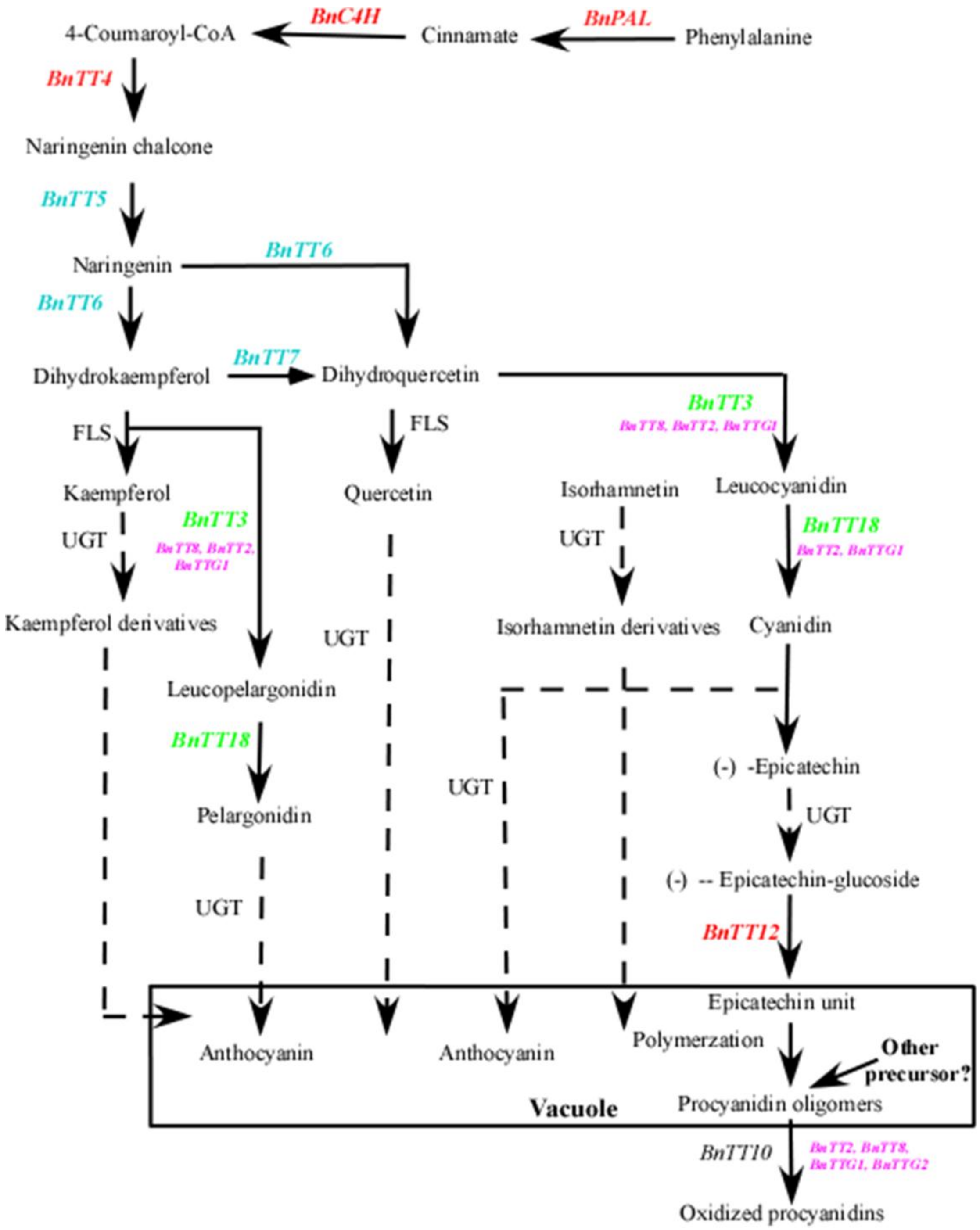

Supplementary Figure S3 The pathway of flavonoid biosynthesis, modification, and transport in *B. napus*. *BnPAL*, l-phenylalanine ammonialyase; FLS, flavonol synthase; *BnC4H*, cinnamate 4-hydroxylase; UGT, UDP flavonoid glucosyl transferase.
